# Real-time hydrogen molecular dynamics satisfying the nuclear spin statistics of a quantum rotor

**DOI:** 10.1038/s42004-022-00788-z

**Published:** 2022-12-03

**Authors:** Kim Hyeon-Deuk, I-Ya Chang

**Affiliations:** Department of Chemistry, Kyoto University, Kyoto, 606-8502 Japan

**Keywords:** Chemical physics, Molecular dynamics

## Abstract

Apparent presence of the nuclear-spin species of a hydrogen molecule, para-hydrogen and ortho-hydrogen, associated with the quantum rotation is a manifestation of the nuclear quantum nature of hydrogen, governing not only molecular structures but also physical and chemical properties of hydrogen molecules. It has been a great challenge to observe and calculate real-time dynamics of such molecularized fermions. Here, we developed the non-empirical quantum molecular dynamics method that enables real-time molecular dynamics simulations of hydrogen molecules satisfying the nuclear spin statistics of the quantum rotor. While reproducing the species-dependent quantum rotational energy, population ratio, specific heat, and H-H bond length and frequency, we found that their translational, orientational and vibrational dynamics becomes accelerated with the higher rotational excitation, concluding that the nuclear quantum rotation stemmed from the nuclear spin statistics can induce various kinds of dynamics and reactions intrinsic to each hydrogen species.

## Abstract (web summary)

Indistinguishable hydrogen nuclei exhibiting long-time and large-scale collision and repulsion dynamics are inherently hard to accurately describe. Here, the authors develop a non-empirical quantum molecular dynamics method that enables real-time molecular dynamics simulations of hydrogen molecules with nuclear quantum rotation satisfying the nuclear spin statistics of the quantum rotor.

## Introduction

A hydrogen molecule H_2_, the simplest of all molecular species, has been fundamental to the establishment of quantum mechanics and quantum chemistry. Since the indistinguishability of two fermion protons requires the antisymmetry on the proton interchange^1^, the total nuclear wave function must be symmetric and antisymmetric with the anti-parallel and parallel nuclear spins, respectively, even in the absence of a direct coupling between nuclear spins and rotational degrees of freedom^2,3^. This is the restriction on the nuclear quantum rotation stemmed from the nuclear spin statistics. It is a remarkable property that a hydrogen molecule is specifically classified into two distinct nuclear-spin species called para-hydrogen and ortho-hydrogen with respect to the symmetric and antisymmetric nuclear rotational wave function related with even and odd rotational quantum numbers, respectively^4–6^. Such nuclear quantum rotation is purely quantum and should be distinguished from a classical rotational motion of a molecular axis^7^. The apparent presence of these nuclear-spin species associated with the nuclear quantum rotation is a manifestation of the nuclear quantum nature of hydrogen, governing not only molecular structures but also physical and chemical properties of hydrogen molecules^8–10^. Therefore, para-hydrogen and ortho-hydrogen have attracted broad interest and showed interdisciplinary importance in species-dependent chemical reactions^11,12^, high-resolution nuclear magnetic resonance^13,14^, molecular superfluidity^15^, formation and evolution of interstellar matters^16–18^, and hydrogen liquefaction and storage as carbon-neutral energy source^19^.

Separately probing and distinguishing para-hydrogen and ortho-hydrogen have been a challenging task^3,20,21^. Theoretically, static nuclear-electron wave functions of para-hydrogen and ortho-hydrogen optimized at 0 K have been obtained by a complex numerical solution composed of more than 100 nuclear and electron basis terms in vacuum^2,22–25^ and also in small nano cages with the reproduction of inelastic neutron scattering spectra using the static and numerical eigenvectors and eigenvalues^26–35^. Such complicated nuclear-electron wave functions are not feasible for real-time molecular dynamics of para-hydrogen and ortho-hydrogen. In fact, it has been a great challenge to calculate paired and molecularized nuclear fermion dynamics that follows the nuclear spin statistics of the quantum rotor although various kinds of insights that only real-time molecular dynamics calculations can provide exist^36–38^. Especially, real-time dynamics of ortho-hydrogen with the odd rotational quantum number is computationally difficult to calculate because its antisymmetrized nuclear density matrix, which has been used as a propagator in traditional path integral methods to treat the nuclear quantum nature, has negative contributions due to the Legendre polynomials, and the negative propagator breaks the physical interpretation of the density matrix as a probability measure, causing extremely low efficiency for sampling paths and even diverges^5,39,40^.

Here, we developed the non-empirical quantum molecular dynamics method that enables real-time molecular dynamics simulations of hydrogen molecules satisfying the nuclear spin statistics of the quantum rotor. While reproducing the species-dependent quantum rotational energy, population ratio, specific heat, and H-H bond length and frequency, we found that their translational, orientational and vibrational dynamics becomes accelerated with the higher rotational excitation, concluding that the nuclear quantum rotation stemmed from the nuclear spin statistics can induce various kinds of dynamics and reactions intrinsic to each hydrogen species.

## Results and discussion

### The nuclear and electron wave packet molecular dynamics method with quantum rotation

**Fig. 1:**
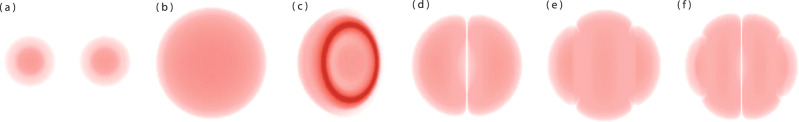
Nuclear distribution of hydrogen molecules. **a** Nuclear distribution of Gaussian without a coupling between nuclear spins and a nuclear rotational state. Nuclear distributions of para (**b**, **c**), ortho (**d**), para-2 (**e**), and ortho-2 (**f**) that follow the nuclear spin statistics of the quantum rotor. The internal shell structure cut in half of para is explicitly shown in (**c**). Nuclear nodes appear in the shell-type species depending on the rotationally-excited states.

We developed the non-empirical quantum molecular dynamics method satisfying the nuclear spin statistics of the quantum rotor called the nuclear and electron wave packet molecular dynamics method with quantum rotation (the NEWPMD-QR method) by extending the Gaussian NEWPMD method (the G-NEWPMD method) that is free from the nuclear spin statistics^41–49^. (See Method and Supplementary Section 1) The nuclear distribution of the stable hydrogen molecule calculated by the G-NEWPMD method exhibited the dumbbell-shape nuclear delocalization composed of the two distinct Gaussian nuclear wave packets (NWPs)^41–49^ (Fig. 1a), and we will call it Gaussian throughout this Article. The NEWPMD-QR method describes two nuclei, A and B, by NWPs via the time-dependent Hartree approach, while it expresses two electrons, L and S, by two Gaussian electron wave packets (EWPs) through the perfect-pairing (PP) valence bond (VB) theory that appropriately treats the Pauli exclusion energy. We derived the time-dependent nuclear-electron wave function that satisfies the nuclear spin statistics of the quantum rotor; it is symmetric and antisymmetric with the anti-parallel and parallel nuclear spins, respectively, with the spherical harmonics *Y*_*Jm*_(*χ*, *ω*) for para (*J* = 0), ortho (*J* = 1), para-2 (*J* = 2), and ortho-2 (*J* = 3). (See Eq. (1) in Methods). While the nuclear distribution of para is a completely spherical shell (Fig. 1b, c), the nuclear distributions of ortho (Fig. 1d), para-2 (Fig. 1e), and ortho-2 (Fig. 1f) exhibit one, two and three nuclear nodes reflecting the spherical harmonics, respectively. It should be also emphasized that the time-dependent nuclear-electron wave function can be derived only when the NWP width of A and B is identical, i.e., Ω_A_ = Ω_B_, which gives the important physical insight that the current formulation for the nuclear quantum rotation is valid only for the two indistinguishable nuclei, i.e., A = B. If the NWP width of the two hydrogen nuclei were different, i.e., Ω_A_ ≠ Ω_B_, the two hydrogen nuclei would be distinguishable and thus could not satisfy the nuclear spin statistics of the quantum rotor for indistinguishable fermions. The simple and analytical nuclear-electron wave function adopted in the NEWPMD-QR method enables real-time dynamics calculations of hydrogen molecules satisfying the nuclear spin statistics of the quantum rotor, which shouldbe distinguished from the previous complex and numerical nuclear-electron wave function composed of more than 100 nuclear and electron bases^2,22–25^. The NEWPMD-QR method is the computational method to calculate real-time molecular dynamics on a nuclear excited state not on an electronically excited state. We started collision dynamics in a single-walled carbon nano tube (SWCNT), CNT(15,0), with the same initial velocity but with the different initial angles between the molecular axis and the CNT(15,0) surface set as 0 degree, 45 degree, and 90 degree which will be called CNT-0, CNT-45, and CNT-90 in this Article, respectively.

### Molecular Structure and Energy in Nuclear Excited States

**Fig. 2:**
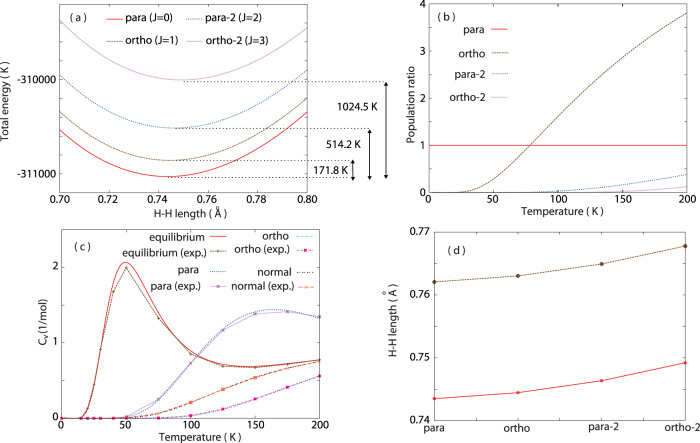
Energy and structure of para, ortho, para-2, and ortho-2. **a** Total molecular energy as a function of the H-H bond length *R*_rel_ with the difference of the minimum molecular energy estimated from the basins of the total energy curves. **b** Population ratios of the shell-type species as a function of temperature relative to the para population in a dilute gas phase. **c** Rotational specific heat at constant volume, *C*_v_, as a function of temperature for the equilibrium population ratio shown in (**b**) (red line), for the para and para-2 population only (blue line), for the ortho and ortho-2 population only (light blue line), and for the normal population ratio composed of 25 %*C*_v_ of para and para-2 and 75 %*C*_v_ of ortho and ortho-2 (black line). The corresponding results obtained from the experimental data are also shown as points with a line for comparison. **d** Stable H-H bond length at the energy minima in (**a**) (red line) as well as the quantum H-H bond length taking into account the NWP delocalization (brown line).

The NEWPMD-QR method reproduces various experimental data without any empirical parameter in spite of the simple and analytical nuclear-electron wave function. The total molecular energy curves with the higher rotational excitation locate at the higher energy region in the order of ortho-2, para-2, ortho and para. (Fig. 2a) At the minimum of each energy curve, the stable hydrogen molecule is formed in each rotationally-excited state. (Fig. 1b–f) The minimum energy rises as *J* increases reflecting the nuclear rotational energy of each species. (Fig. 2a and Supplementary Table 1) The energy gaps, which are 171.8 K, 514.2 K and 1024.5 K between para and ortho (*E*_o,p_), para-2 (*E*_p2,p_) and ortho-2 (*E*_o2,p_), are in the good agreement with the experimental data^2,22^. (Supplementary Table 1) The rotational constant B directly estimated from *E*_o,p_ = 2*B* as *B* = 85.9 K is also close to the experimental value *B* = 85.4 K^2^.

The population ratios of each species relative to the para population in a dilute gas phase was calculated using the expressions *P*_p_ = 1 (para), ${P}_{{{{{{{{\rm{o}}}}}}}}}=9\exp (-{{\Delta }}{E}_{{{{{{{{\rm{o}}}}}}}},{{{{{{{\rm{p}}}}}}}}}/T)$ (ortho), ${P}_{{{{{{{{\rm{p}}}}}}}}2}=5\exp (-{{\Delta }}{E}_{{{{{{{{\rm{o}}}}}}}}2,{{{{{{{\rm{p}}}}}}}}}/T)$ (para-2), and ${P}_{{{{{{{{\rm{o}}}}}}}}2}=21\exp (-{{\Delta }}{E}_{{{{{{{{\rm{p}}}}}}}}2,{{{{{{{\rm{p}}}}}}}}}/T)$ (ortho-2). (Fig. 2b) The numerical prefactors in the ratio expressions correspond to the species-dependent degeneracy^50^. The para ratio is larger than 95% even at the critical temperature, 33 K, and still dominant. However, the ortho ratio exceeds the para ratio around 78.2 K, and finally becomes dominant at 200 K. The ratios of the higher-rotational species also become significant in the higher-temperature region; the para-2 ratio appears around 100 K while the ortho-2 ratio can be seen only above 150 K. Therefore, the rotationally-excited species, ortho, para-2, and ortho-2, cannot be neglected in the higher-temperature region. The ratio of the para species (para and para-2) over the ortho species (ortho and ortho-2) is calculated as 0.351 at 200 K (Supplementary Table 1), approaching to the ratio of normal hydrogen, 0.333^8^.

The different characters of the para and ortho species most significantly influence high-temperature thermophysical properties like the specific heat^51^. The energy difference accompanying the population ratios enable calculations of the rotational specific heat *C*_*v*_ for equilibrium hydrogen (*E*_o,p_*P*_o_ + *E*_p2,p_*P*_p2_ + *E*_o2,p_*P*_o2_)/(*P*_p_ + *P*_o_ + *P*_p2_ + *P*_o2_), para-hydrogen (*E*_p2,p_*P*_p2_)/(*P*_p_ + *P*_p2_), ortho-hydrogen (*E*_o,p_*P*_o_ + *E*_o2,p_*P*_o2_)/(*P*_o_ + *P*_o2_), and normal hydrogen composed of 25 % para-hydrogen and 75 % ortho-hydrogen^8,50^. (Fig. 2c) The rotational specific heat calculated by the NEWPMD-QR method shows the excellent agreements with the experimental data without any fitting parameter^50^: The NEWPMD-QR method well reproduced that the *C*_v_-peaks appear only in the equilibrium hydrogen and para-hydrogen cases and no *C*_v_-peak can be found in the ortho-hydrogen and normal hydrogen cases.

The stable H-H bond length *R*_rel_ for each species can be determined from the energy minimum in Fig. 2a. (The red line in Fig. 2d) We found that the quantum H-H length in which the nuclear delocalization is taken into consideration becomes longer than the H-H length by 2.5%. (The brown line in Fig. 2d) In the both cases, the H-H bond length becomes elongated on the higher rotational state–the higher-energy nuclear rotation induces the stronger centrifugal force inside the molecule, leading to the longer H-H length. (Supplementary Table 2) The experimental data for the H-H length, defined as the minimum of the intramolecular potential, is known as 0.741 Å^2^ that is close to the calculated para H-H length (0.74349 Å) and to the calculated Gaussian H-H length (0.74551 Å)^41–49^. The experimental data for the quantum H-H length, defined as a mean value of the intramolecular distance for the lowest vibrational state, was reported as 0.752 Å^52^. This is again close to the calculated quantum H-H length of para (0.76207 Å) and of Gaussian (0.76575 Å). The different molecular structures, which are solely specified with the NWP center position and width (Fig. 2d, Supplementary Fig. 1, Supplementary Fig. 2a and Supplementary Table 2) as well as the EWP width and center position (Supplementary Fig. 2b, c), Supplementary Fig. 3, Supplementary Fig. 4 and Supplementary Table 3), and the different energies between the G-NEWPMD and NEWPMD-QR methods can be solely attributed to the different nuclear spin statistics (See Method); the NEWPMD-QR method follows the quantum statistics of the Fermi rotor and the nuclear spin and rotation are closely coupled although the G-NEWPMD method is free from such restriction.

### Molecular Dynamics on Nuclear Excited States

Encouraged by the sufficient accuracy of the NEWPMD-QR method demonstrated in Fig. 2, we calculated real-time collision dynamics of a hydrogen molecule occurring inside CNT(15,0). The real-time collision dynamics of the shell-type species (para, ortho, para-2, and ortho-2) on the rotationally excited states was visualized showing the species-dependent delocalized nuclei and nodes in Supplementary Movies 1-5. Such real-time movies could not be obtained by a static nuclear-electron wave function optimized at 0 K or by any computational method in which the nuclear spins and quantum rotational states are not coupled. These movies clearly show how a hydrogen molecule possessing a multipole composed of delocalized nuclei and electrons acts in a laboratory coordinate system during real-time collision dynamics.

**Fig. 3:**
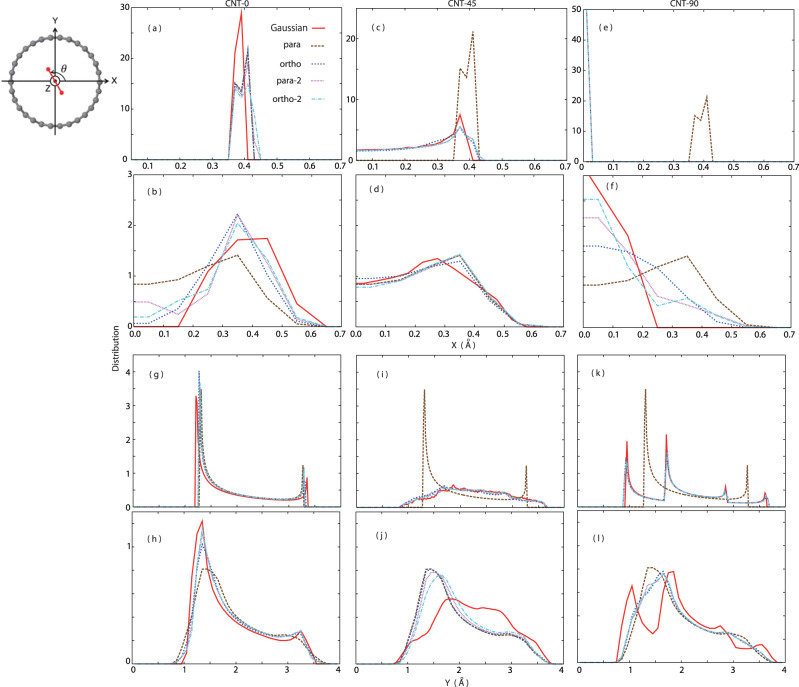
Spatial distributions of a hydrogen molecule achieved by the collision dynamics taking place inside CNT(15,0). Spatial distributions of Gaussian, para, ortho, para-2, and ortho-2 along the *x*-axis (**a**–**f**) and along the *y*-axis (**g**–**l**). The initial collision angles between the molecular axis and the CNT(15,0) surface are set as 0 degree (CNT-0: **a**, **b**, **g**, **h**), 45 degree (CNT-45: **c**, **d**, **i**, **j**), and 90 degree (CNT-90: **e**, **f**, **k**, **l**). Panels in the first and third rows show distributions of the NWP center positions **R**_A_(*t*) and **R**_B_(*t*) along the *x*- and *y*-axes, respectively. Panels in the second and fourth rows display corresponding quantum distributions considering the species-dependent NWP delocalization along the *x*- and *y*-axes, respectively.

The *X*-distributions of the NWP center positions **R**_A_(*t*) and **R**_B_(*t*), which are introduced in Supplementary Eq.(6), in CNT-0 exhibit two split peaks corresponding to the shortest and longest H-H bond during the H-H vibration although the two peaks are too close to be clearly separated in Gaussian. (Fig. 3a) Their quantum *X*-distributions that takes account of the species-dependent NWP delocalization becomes much broader. (Fig. 3b) The quantum *X*-distribution for para (the brown line in Fig. 3b) expresses a projection of the perfectly spherical nuclear shell of para on the *x*-axis. The dents of the quantum *X*-distributions around *X* = 0.0 Å are deeper in the order of Gaussian, ortho, ortho-2, para-2, and para reflecting the nuclear density and nodes around the molecular center. Because the collision dynamics in CNT-45 induces various kinds of orientational dynamics, the *X*-distributions of CNT-45 become more uniform and similar regardless of the species except for para. (Fig. 3c) Since para can be treated as a completely spherical particle, there is no need to sample its orientation, and we adopted the same trajectory for para in any collision angle–this is the notable feature of para. However, all the quantum *X*-distributions of CNT-45 become almost overlapped including the para case due to its broad nuclear delocalization, indicating the importance of the nuclear delocalization to obtain a correct spatial distribution of para. (Fig. 3d) The role of the nuclear delocalization is most significant in CNT-90. (Fig. 3e, f) Although all the *X*-distributions are a simple delta function except for para, the quantum *X*-distributions have the finite broadness due to the delocalized nuclei. Because Gaussian does not have significant nuclear delocalization along the *x*-axis in CNT-90, the broadness of the quantum *X*-distribution of Gaussian is almost half of the quantum *X*-distributions of the shell-type species in CNT-90.

Each *Y*-distribution of the NWP center positions **R**_A_(*t*) and **R**_B_(*t*) in CNT-0 has two sharp peaks at the both sides (Fig. 3g); the higher peak corresponds to a long stay around the CNT center where the hydrogen molecule has relatively low translational velocity along the *y* -axis due to the gentle potential energy curve. (Supplementary Fig. 5) The lower peak indicates the short-time physical adsorption and desorption of the hydrogen molecule to the CNT(15,0) surface because of the steep interaction potential energy curve around the surface (Supplementary Fig. 5); the hydrogen molecule bounces off the CNT(15,0) surface within a short time as Supplementary Movies 1-5 display. The sharp distribution peaks are rounded in the quantum *Y*-distributions due to the significant nuclear delocalization. (Fig. 3h) Various kinds of the orientational dynamics taking place in CNT-45 again leads to the more uniform and similar *Y*-distributions for all the species other than para. (Fig. 3i) However, the difference between Gaussian and the shell-type species becomes obvious in the quantum *Y*-distributions of CNT-45 due to the distinct dumbbell-type nuclear delocalization of Gaussian. (Fig. 3j) Except for para, each of the *Y*-distributions in CNT-90 has four sharp peaks decomposed into the upper and lower sets of the paired two peaks; each set corresponds to the paired two hydrogen nuclei forming one hydrogen molecule. (Fig. 3k) Actually, the split distance between the paired peaks in both the upper and lower sets is approximately 0.74 Å close to the H-H bond length. Such split peaks are smeared out to one broad peak in the quantum *Y*-distributions of the shell-type species by the broad nuclear delocalization although the quantum *Y*-distribution of Gaussian still exhibits the two split peaks reflecting its dumbbell shape. (Fig. 3l)

On one hand, the spatial distribution made from the collision dynamics of Gaussian free from the restriction on the nuclear quantum rotation is highly sensitive to the collision angle. On the other hand, the spatial distributions achieved by the collision dynamics of the shell-type species that follows the nuclear spin statistics of the quantum rotor are much less sensitive to the collision angle and they are similar regardless of the species. Our finding that the translational dynamics smeared out the difference in the nuclear distribution and nodes of the shell-type species could be the reason why para- and ortho-hydrogens have the similarity in many physical and chemical properties^8,9^.

**Fig. 4:**
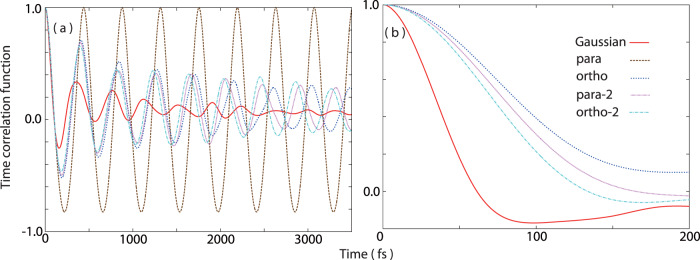
Real-time translational and orientational dynamics of hydrogen molecules during the collision with CNT(15,0). **a** TCFs of the position of the COM **R**_COM_(*t*) and **b** TCFs of the time-dependent angle *θ*(*t*) between the molecular axis and the CNT(15,0) surface as a function of time in CNT-45. The *θ*-TCF for para is omitted because para is completely spherical and does not have any orientational degree of freedom.

The NEWPMD-QR method further provides new insights to real-time translational and orientational dynamics on the rotationally-excited states. Although the quantum *X*- and *Y*-distributions of CNT-45 are almost overlapped in any species (Fig. 3d, j), their real-time translational dynamics described by a time-correlation function (TCF) of the position of the center-of-mass (COM) **R**_COM_(*t*) deviates significantly as time goes by. (Fig. 4a) The TCF of Gaussian decays fastest compared to the TCFs of the shell-type species because Gaussian moves most actively due to its dumbbell shape. In contrast, the TCF of para keeps stably oscillating due to its completely spherical shape. For the other shell-type species, the oscillating speed of the TCFs is faster with the larger *J* in the order of ortho-2, para-2 and ortho; the less spherical and more anisotropic molecular shapes induced by the higher-energy quantum rotation accelerate the translational collision dynamics even with the same initial velocity and collision angle.

Although the orientational distributions of CNT-45 are uniform and similar in the all species studied (Supplementary Fig. 6), their real-time orientational dynamics also differs clearly. (Fig. 4b) The *θ*-TCF of Gaussian exhibits the most rapid decay because the dumbbell shape of Gaussian induces more active orientational dynamics than the shell-type species, which is one of the notable properties of dumbbell-shape molecules without the coupling between the nuclear spin and quantum rotational states. The *θ*-TCFs of the shell-type species decay more rapidly with the higher-level rotational excitation, that is, in the order of ortho-2, para-2 and ortho; the stronger anisotropy of the molecular structure with the higher-energy quantum rotation promotes not only the translational dynamics but also the orientational dynamics even with the same initial velocity and collision angle. In fact, the completely spherical para, which does not have any rotational degree of freedom, does not have any orientational dynamics. Figure 4 shows the evidence that the nuclear spin statistics and the resulting nuclear quantum rotation affect the real-time translational and orientational dynamics of hydrogen molecules, which could not be found by a conventional static nuclear-electron wave function.

**Fig. 5:**
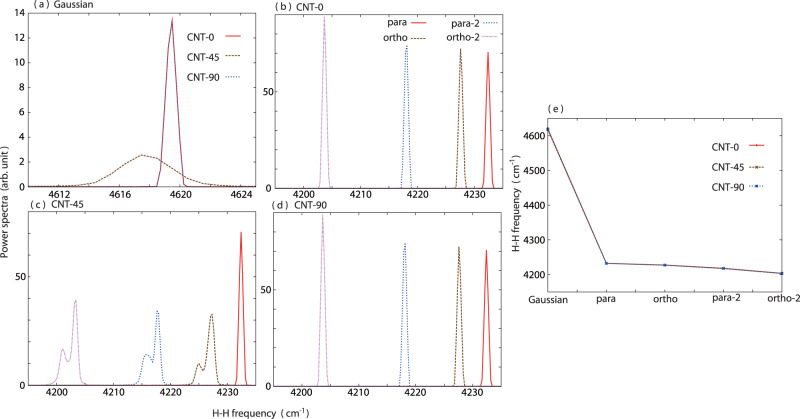
H-H vibrational dynamics during the collision dynamics with CNT(15,0). Vibrational power spectra of the H-H bond length *R*_rel_(*t*) of Gaussian (**a**) for the initial collision angles 0 degree (CNT-0), 45 degree (CNT-45), and 90 degree (CNT-90). Vibrational power spectra of the H-H bond length *R*_rel_(*t*) of the shell-type species for CNT-0 (**b**), CNT-45 (**c**), and CNT-90 (**d**). **e** H-H vibrational frequencies estimated from the peak frequencies of the vibrational power spectra shown in (**a**–**d**).

Intramolecular dynamics, the H-H vibration in the collision dynamics with CNT(15,0), is another key dynamics that can be directly calculated by the NEWPMD-QR method. An H-H vibrational power spectrum reflects the complex translational and orientational dynamics of the hydrogen molecule on the anharmonic interaction potential energy with the CNT(15,0). The H-H vibrational power spectra of Gaussian in CNT-0 and CNT-90 are sharp and identical (Fig. 5a); the H-H bond of Gaussian is stiff enough not to be affected by the simple straight collisions. Meanwhile, as demonstrated in Fig. 4, the most active translational and orientational dynamics induced in CNT-45 due to the dumbbell shape of Gaussian leads to the much broader power spectrum than any vibrational power spectra of the shell-type species shown in Fig. 5b–d. The H-H vibrational power spectra of the shell-type species in CNT-0, CNT-45 and CNT-90 are red-shifted with the higher-energy nuclear rotation; the extent of the red shift is in the order of ortho-2, para-2, ortho and para. (Fig. 5b–d) The higher-energy quantum rotation inducing the stronger centrifugal force and more nuclear nodes makes the H-H bond longer and weaker (Fig. 2d), leading to the larger red shift of the H-H vibration. The weaker H-H bond caused by the higher-level rotational excitation results in the larger intensity of the H-H vibrational power spectra in the same order of the red shift except for the para spectrum in CNT-45; para is free from the collision angle. It is remarkable that all of the above changes found in the H-H vibrational power spectra originate not from the electronic excitation but purely from the nuclear excitation.

The H-H vibrational power spectra for the same species in CNT-0 and CNT-90 are sharp and identical (Fig. 5b, d); the H-H bonds of the shell-type species are so stiff that the spectral shapes and frequencies are not affected by the simple straight collisions. In contrast, the H-H vibrational power spectra of ortho, para-2 and ortho-2 in CNT-45 become broader and have two split peaks. (Fig. 5c) Various kinds of the collision dynamics generated in CNT-45 disperse the H-H vibration and broadened the H-H vibrational power spectra, decreasing the intensity. The lower-frequency peak of the two split peaks corresponds to the H-H vibration under a strong trap to the CNT wall; the H-H bond is weakened and the H-H frequency is red-shifted due to the strong trapping to the CNT wall. In fact, the higher-frequency peak corresponding to the H-H vibration without the strong trapping has the frequency close to the peak frequency in CNT-0 and CNT-90. Because the adsorption and desorption time is short in the current collision dynamics and also because the H-H vibrational amplitude is suppressed due to the restricted space near the CNT wall, the intensity of the lower-frequency peak is smaller than the intensity of the higher-frequency peak. Such dynamical power spectra exhibiting the complicated peak shapes and intensity as well as the TCF of the H-H vibration (Supplementary Fig. 7) cannot be obtained by a quantum chemistry calculation using a static Hessian matrix or by any computational method in which the nuclear spin and rotational states are not coupled.

The H-H vibrational frequencies are almost the same regardless of the initial collision angle of CNT-0, CNT-45 and CNT-90, being in the good agreements with the experimental data^22,51^; e.g. 4232.410 cm^−1^ for para, 4227.574 cm^−1^ for ortho, 4218.158 cm^−1^ for para-2, and 4203.652 cm^−1^ for ortho-2 in CNT-0. (Fig. 5e) and Supplementary Table 4) It is notable that the experimental vibrational frequencies were reproduced within the 1.9% deviation by the NEWPMD-QR method adopting the simple and analytical time-dependent nuclear-electron wave function that is feasible even in a real-time molecular dynamics simulation. In contrast, the G-NEWPMD method overestimated the experimental H-H vibrational frequency as 4619.488 cm^−1^ in CNT-0; the deviation is approximately 11.0%. (Supplementary Table 4) The great improvement of the H-H vibrational frequency achieved in the NEWPMD-QR method is solely attributed to the nuclear spin statistics of the quantum rotor introduced in the NEWPMD-QR method. In other words, it was demonstrated that not only the electronic state but also the nuclear quantum rotation coupling to the nuclear spin state actually influence the molecular vibrational dynamics and its frequencies^10^.

## Conclusions

The NEWPMD-QR method satisfying the nuclear spin statistics of the quantum rotor enabled us to calculate the real-time molecular dynamics of the hydrogen molecules composed of the indistinguishable hydrogen nuclei on the different quantum rotational states such as long-time and large-scale collision and repulsion dynamics including adsorption and desorption dynamics inside CNT(15,0), and also allowed the direct comparison with the real-time dynamics of the hydrogen molecule without the restriction on the nuclear quantum rotation. It is surprising that the current simple and analytical nuclear-electron wave function applicable even to real-time dynamics successfully reproduced the experimental observations such as the rotational excitation energy, the rotational constant, the species-dependent population ratio, the temperature-dependent specific heat, and the H-H bond length with the sufficient accuracy. The finding that the real-time translational, orientational and vibrational dynamics clearly differs depending on the nuclear quantum rotation proposes the possibility to dynamically separate hydrogen species of the different quantum rotation, and to detect and extract specific species as the dynamical quantum sieving, which can contribute to the effective hydrogen liquefaction and storage as the carbon-neutral energy source^19,53^. The different collision dynamics taking place inside CNT(15,0) demonstrated here also strongly indicates that various kinds of dynamics and reactions can be actually generated depending on the hydrogen species on a carbon surface in space, influencing and differentiating formation and evolution of interstellar matters^16–18^. The insight that the nuclear quantum rotation associated with the nuclear spin statistics plays a pivotal role in real-time molecular dynamics of a hydrogen molecule could not be obtained by a conventional calculation of a static and optimized nuclear-electron wave function or by any computational method in which the nuclear spins and quantum rotational states are not coupled.

Actually, any computational method to obtain a static, numerical, and optimized nuclear-electron wave function itself does not describe a time-evolving quantity and thus can not produce the main results shown in Figs. 3–5, Supplementary Figs. 6, 7, and Supplementary Table 4, which were obtained by time-averaging 500 ps real-time trajectories of time-evolving nuclear-electron wave functions of hydrogen molecules with different nuclear quantum rotation, as well as Supplementary Movies 1–5. For example, in Fig. 3c, d, i, j, if we did not time-evolve an initial nuclear-electron wave function of a hydrogen molecule, the shown spatial distributions of a hydrogen molecule time-averaged over 500 ps collision and repulsion trajectories would not be obtained since the initial collision angle between the H-H molecular axis and the CNT(15,0) surface set as *θ*(0) = 45 degree would be simply optimized to the most stable angle *θ*(0) = 90 degree without any time evolution. The TCFs shown in Fig. 4 are clearly dynamical since they are a function of time, and thus cannot be obtained by previous time-independent nuclear-electron wave function optimized at 0 K. The dynamical power spectra shown in Fig. 5 exhibit the complicated peak shapes and intensity reflecting complex real-time collision and repulsion dynamics including adsorption and desorption dynamics inside CNT(15,0), and can not be obtained by a quantum chemistry calculation using a static Hessian matrix.

Introducing hydrogen molecules inside a CNT has been widely studied experimentally and computationally^54,55^. It is possible to experimentally make a similar situation to the current computational situation by introducing dilute hydrogen gas into a CNT. Inelastic neutron scattering spectra and infrared vibrational spectra can probe the details^56,57^.

## Methods

### Time-dependent nuclear-electron wave function satisfying the nuclear spin statistics of a quantum rotor

The time-dependent nuclear-electron wave function for a hydrogen molecule composed of the two nuclei A and B and the two electrons L and S with the rotational quantum number *J* is introduced as ${{{\Psi }}}_{{{{{{{{\rm{AB}}}}}}}},J}^{{{{{{{{\rm{L,S}}}}}}}}}(t)={{{\Phi }}}_{{{{{{{{\rm{AB}}}}}}}},J}({{{{{{{{\bf{Q}}}}}}}}}_{1},{{{{{{{{\bf{Q}}}}}}}}}_{2},t){\psi }_{{{{{{{{\rm{L,S}}}}}}}}}({{{{{{{{\bf{q}}}}}}}}}_{1},{{{{{{{{\bf{q}}}}}}}}}_{2},t)$, where **Q**_1_, **Q**_2_, **q**_1_ and **q**_2_ denote A-, B-, L- and S-coordinates, respectively. (Supplementary Section 1A) The normalized time-dependent PP VB electronic wave function for the two electrons *ψ*_L,S_(**q**_1_, **q**_2_, *t*) has the same form as the G-NEWPMD method’s except for the EWP width and its dependence on the H-H bond length. (Supplementary Section 1B) We first derived such time-dependent nuclear wave function that satisfies the nuclear spin statistics of the quantum rotor; it is symmetric and antisymmetric with the anti-parallel and parallel nuclear spins, respectively, as

1 $${{{\Phi }}}_{{{{{{{{\rm{AB}}}}}}}},J}({{{{{{{{\bf{Q}}}}}}}}}_{1},{{{{{{{{\bf{Q}}}}}}}}}_{2},t)=	\, {N}_{{{{{{{{\rm{AB}}}}}}}},J}^{-1}\exp \left[-\frac{{({{{{{{{{\bf{Q}}}}}}}}}_{{{{{{{{\rm{COM}}}}}}}}}-{{{{{{{{\bf{R}}}}}}}}}_{{{{{{{{\rm{COM}}}}}}}}}(t))}^{2}}{2{{{\Omega }}}_{{{{{{{{\rm{A}}}}}}}}}^{2}(t)}-\frac{{({Q}_{{{{{{{{\rm{rel}}}}}}}}}-{R}_{{{{{{{{\rm{rel}}}}}}}}}(t))}^{2}}{8{{{\Omega }}}_{{{{{{{{\rm{A}}}}}}}}}^{2}(t)}\right.\\ 	+{{{{{{{\rm{i}}}}}}}}\frac{{{{\Pi }}}_{{{{{{{{\rm{A}}}}}}}}}(t)}{{{{\Omega }}}_{{{{{{{{\rm{A}}}}}}}}}(t)}{({{{{{{{{\bf{Q}}}}}}}}}_{{{{{{{{\rm{COM}}}}}}}}}-{{{{{{{{\bf{R}}}}}}}}}_{{{{{{{{\rm{COM}}}}}}}}}(t))}^{2}+{{{{{{{\rm{i}}}}}}}}\frac{{{{\Pi }}}_{{{{{{{{\rm{A}}}}}}}}}(t)}{4{{{\Omega }}}_{{{{{{{{\rm{A}}}}}}}}}(t)}{({Q}_{{{{{{{{\rm{rel}}}}}}}}}-{R}_{{{{{{{{\rm{rel}}}}}}}}}(t))}^{2}\\ 	\left.+2{{{{{{{\rm{i}}}}}}}}{{{{{{{{\bf{P}}}}}}}}}_{{{{{{{{\rm{COM}}}}}}}}}(t)\cdot ({{{{{{{{\bf{Q}}}}}}}}}_{{{{{{{{\rm{COM}}}}}}}}}-{{{{{{{{\bf{R}}}}}}}}}_{{{{{{{{\rm{COM}}}}}}}}}(t))+{{{{{{{\rm{i}}}}}}}}\frac{{P}_{{{{{{{{\rm{rel}}}}}}}}}(t)}{2}({Q}_{{{{{{{{\rm{rel}}}}}}}}}-{R}_{{{{{{{{\rm{rel}}}}}}}}}(t))\right]{Y}_{Jm}(\chi ,\omega ),$$

with the *J*-dependent normalization factor *N*_AB,*J*_. The COM and relative coordinates, **Q**_COM_ = (**Q**_1_ + **Q**_2_)/2 and **Q**_rel_ = **Q**_1_ − **Q**_2_, were introduced to effectively describe the nuclear quantum rotation with the COM position **R**_COM_(*t*) and the relative position **R**_rel_(*t*) defined by the distance between the two NWP center positions, and their conjugate momenta **P**_COM_(*t*) and **P**_rel_(*t*), respectively. Ω_A_(*t*) and Π_A_(*t*) are the NWP width and its conjugate momentum, respectively. The spherical harmonics *Y*_*Jm*_(*χ*, *ω*) with three-dimensional angular coordinates *χ* and *ω* expresses the angular wave function for para (*J* = 0), ortho (*J* = 1), para-2 (*J* = 2), and ortho-2 (*J* = 3). Their stable nuclear distributions, ${{{\Phi }}}_{{{{{{{{\rm{AB}}}}}}}},J}^{* }({{{{{{{\bf{Q}}}}}}}},{{{{{{{\bf{Q}}}}}}}},t){{{\Phi }}}_{{{{{{{{\rm{AB}}}}}}}},J}({{{{{{{\bf{Q}}}}}}}},{{{{{{{\bf{Q}}}}}}}},t)$, all have the shell-type nuclear distributions owing to the rotational wave function, $\exp [-{({Q}_{{{{{{{{\rm{rel}}}}}}}}}-{R}_{{{{{{{{\rm{rel}}}}}}}}}(t))}^{2}/8{{{\Omega }}}_{{{{{{{{\rm{A}}}}}}}}}^{2}(t)]$. The stable nuclear distributions displayed in Fig. 1b–f correspond to ${{{\Phi }}}_{{{{{{{{\rm{AB}}}}}}}},J}^{* }({{{{{{{\bf{Q}}}}}}}},{{{{{{{\bf{Q}}}}}}}},t){{{\Phi }}}_{{{{{{{{\rm{AB}}}}}}}},J}({{{{{{{\bf{Q}}}}}}}},{{{{{{{\bf{Q}}}}}}}},t)$ at the minimum of *E*_tot,*J*_ for each *J* shown in Fig. 2a. In the classical limit of a rigidly oriented molecule, where the nuclear spin state is not coupled to the quantum rotational state, the angular part becomes a simple delta-function *δ*(*χ*) which is an infinite sum over the all spherical harmonics ∑_*J*,*m*_*Y*_*Jm*_(*χ*, *ω*), meaning that all rotational states are equally excited and mixed in the classical-rotation limit^7^.

### Kinetic energy and electrostatic interaction energy

The total molecular energy *E*_tot,*J*_ is a sum of the kinetic energy of the hydrogen nuclei and electrons, and the three electrostatic interaction energy of electron-electron, nucleus-nucleus and nucleus-electron. Because the electronic wave function for the two electrons, *ψ*_L,S_(**q**_1_, **q**_2_, *t*), is common in the NEWPMD-QR and G-NEWPMD methods^41–49^, the kinetic energy of the two electrons *E*_ke,elec_ and the electron-electron interaction energy *E*_ee_ have the same forms in the both methods. In contrast, The kinetic energy of the hydrogen nuclei depending on the rotational quantum number *J* derived in the NEWPMD-QR method is

2 $${E}_{{{{{{{{\rm{ke,nuc}}}}}}}},J}=	\, \frac{{{{{{{{{\bf{P}}}}}}}}}_{{{{{{{{\rm{COM}}}}}}}}}^{2}(t)}{{M}_{{{{{{{{\rm{nuc}}}}}}}}}}+\frac{{P}_{{{{{{{{\rm{rel}}}}}}}}}^{2}(t)}{4{M}_{{{{{{{{\rm{nuc}}}}}}}}}}\\ 	\, +\frac{2{R}_{{{{{{{{\rm{rel}}}}}}}}}(t){{{\Omega }}}_{{{{{{{{\rm{A}}}}}}}}}(t){P}_{{{{{{{{\rm{rel}}}}}}}}}(t){{{\Pi }}}_{{{{{{{{\rm{A}}}}}}}}}(t)}{{M}_{{{{{{{{\rm{nuc}}}}}}}}}({R}_{{{{{{{{\rm{rel}}}}}}}}}^{2}(t)+2{{{\Omega }}}_{{{{{{{{\rm{A}}}}}}}}}^{2}(t))}+\frac{2{{{\Pi }}}_{{{{{{{{\rm{A}}}}}}}}}^{2}(t)({R}_{{{{{{{{\rm{rel}}}}}}}}}^{2}(t)+3{{{\Omega }}}_{{{{{{{{\rm{A}}}}}}}}}^{2}(t))}{{M}_{{{{{{{{\rm{nuc}}}}}}}}}({R}_{{{{{{{{\rm{rel}}}}}}}}}^{2}(t)+2{{{\Omega }}}_{{{{{{{{\rm{A}}}}}}}}}^{2}(t))}\\ 	+\frac{{R}_{{{{{{{{\rm{rel}}}}}}}}}^{2}(t)+(2J(J+1)+3){{{\Omega }}}_{{{{{{{{\rm{A}}}}}}}}}^{2}(t)}{2{M}_{{{{{{{{\rm{nuc}}}}}}}}}{{{\Omega }}}_{{{{{{{{\rm{A}}}}}}}}}^{2}(t)({R}_{{{{{{{{\rm{rel}}}}}}}}}^{2}(t)+2{{{\Omega }}}_{{{{{{{{\rm{A}}}}}}}}}^{2}(t))},$$

where *M*_nuc_ is relative mass of the hydrogen nucleus to the electron. The final term leads to the energy difference among the shell-type species (para, ortho, para-2, and ortho-2), suggesting that the difference among the shell-type species can be more apparent as the NWP width Ω_A_(*t*) becomes larger and as the H-H bond length *R*_rel_(*t*) becomes smaller. Interestingly, the electrostatic interaction energy of nucleus-nucleus and nucleus-electron calculated in the NEWPMD-QR method was found to be independent of the shell-type species as,

3 $${E}_{{{{{{{{\rm{nn}}}}}}}}}=\frac{2\exp \left[-\frac{{R}_{{{{{{{{\rm{rel}}}}}}}}}^{2}(t)}{4{{{\Omega }}}_{{{{{{{{\rm{A}}}}}}}}}^{2}(t)}\right]{{{\Omega }}}_{{{{{{{{\rm{A}}}}}}}}}(t)+\sqrt{\pi }{R}_{{{{{{{{\rm{rel}}}}}}}}}(t)\left(1+{{{{{{{\rm{erf}}}}}}}}\left[\frac{{R}_{{{{{{{{\rm{rel}}}}}}}}}(t)}{2{{{\Omega }}}_{{{{{{{{\rm{A}}}}}}}}}(t)}\right]\right)}{2\exp \left[-\frac{{R}_{{{{{{{{\rm{rel}}}}}}}}}^{2}(t)}{4{{{\Omega }}}_{{{{{{{{\rm{A}}}}}}}}}^{2}(t)}\right]{{{\Omega }}}_{{{{{{{{\rm{A}}}}}}}}}(t){R}_{{{{{{{{\rm{rel}}}}}}}}}(t)+\sqrt{\pi }({R}_{{{{{{{{\rm{rel}}}}}}}}}^{2}(t)+2{{{\Omega }}}_{{{{{{{{\rm{A}}}}}}}}}^{2}(t))\left(1+{{{{{{{\rm{erf}}}}}}}}\left[\frac{{R}_{{{{{{{{\rm{rel}}}}}}}}}(t)}{2{{{\Omega }}}_{{{{{{{{\rm{A}}}}}}}}}(t)}\right]\right)},$$

and

4 $${E}_{{{{{{{{\rm{ne}}}}}}}}}=\frac{2{V}_{{{{{{{{\rm{LL}}}}}}}},{{{{{{{\rm{AB}}}}}}}}}+2{V}_{{{{{{{{\rm{SS}}}}}}}},{{{{{{{\rm{AB}}}}}}}}}+4{S}_{{{{{{{{\rm{LS}}}}}}}}}(t){V}_{{{{{{{{\rm{LS}}}}}}}},{{{{{{{\rm{AB}}}}}}}}}}{1+{S}_{{{{{{{{\rm{LS}}}}}}}}}^{2}},$$

with the nucleus-electron integral *V*_LS,AB_ and the overlap integral *S*_LS_ because the radial nucleus-nucleus and nucleus-electron interaction depends only on the internuclear distance *Q*_rel_ being free from the angular coordinates *χ* and *ω*. (Supplementary Section 1B).

### Interaction potential energy between a rotationally-excited hydrogen molecule and carbon nano tube

In order to simulate real-time collision dynamics of the shell-type species with a SWCNT, CNT(15,0) of the diameter *D*_CNT_ = 11.71 Å, we calculated the interaction potential energy *E*_CNT,*J*_(*r*, *θ*) as a function of the distance *r* between the COM of the shell-type species and the CNT(15,0) surface and the angle *θ* between the H-H molecular axis and the CNT(15,0) surface by averaging the total energy function *E*_CNT_(*r*, *χ*_CNT_) over the rotational degrees of freedom as,

5 $${E}_{{{{{{{{\rm{CNT}}}}}}}},J}(r,\theta )=\frac{1}{{N}_{{{{{{{{\rm{CNT}}}}}}}},J}^{2}}\int\nolimits_{0}^{{{{{{{{\rm{\pi }}}}}}}}}\sin {\chi }_{{{{{{{{\rm{CNT}}}}}}}}}d{\chi }_{{{{{{{{\rm{CNT}}}}}}}}}\int\nolimits_{0}^{2\pi }d{\omega }_{{{{{{{{\rm{CNT}}}}}}}}}{E}_{{{{{{{{\rm{CNT}}}}}}}}}(r,{\chi }_{{{{{{{{\rm{CNT}}}}}}}}}){Y}_{Jm}^{2}({\chi }_{{{{{{{{\rm{CNT}}}}}}}}}-\theta ,{\omega }_{{{{{{{{\rm{CNT}}}}}}}}}),$$

with the *J*-dependent normalization factor *N*_CNT,*J*_, and

6 $${E}_{{{{{{{{\rm{CNT}}}}}}}}}(r,{\chi }_{{{{{{{{\rm{CNT}}}}}}}}})=a\left[{\left(\frac{b({\chi }_{{{{{{{{\rm{CNT}}}}}}}}})}{r}\right)}^{6.8}-{\left(\frac{b({\chi }_{{{{{{{{\rm{CNT}}}}}}}}})}{r}\right)}^{5.5}\right],$$

which was obtained by fitting the total energy data of a classical hydrogen molecule possessing two point nuclei and CNT(15,0) calculated by the density functional theory (PBE-D2) method.(Supplementary Fig. 8 and Supplementary Section 1D) While the coefficient *a* = 8250.86 K was found to be almost independent of *χ*_CNT_, the coefficient $b({\chi }_{{{{{{{{\rm{CNT}}}}}}}}})=2.559-{\cos }^{2}{\chi }_{{{{{{{{\rm{CNT}}}}}}}}}/10.80$ becomes a function of *χ*_CNT_. (Supplementary Fig. 9)${Y}_{Jm}^{2}({\chi }_{{{{{{{{\rm{CNT}}}}}}}}}-\theta ,{\omega }_{{{{{{{{\rm{CNT}}}}}}}}})$ corresponds to the probability weight to sample the angular wave function intrinsic to each shell-type species. For instance, para has ${Y}_{00}^{2}({\chi }_{{{{{{{{\rm{CNT}}}}}}}}}-\theta ,{\omega }_{{{{{{{{\rm{CNT}}}}}}}}})=1$, requiring the completely spherical angular sampling^2^. While para exhibits the uniform interaction potential energy along *θ* (Supplementary Fig. 5a, e), the interaction potential energy of the other shell-type species show the clear *θ*-dependence depending on the rotationally excited states.(Supplementary Fig. 5e–h) Such orientational preference indicates that the degeneracy of the rotationally excited states is lifted due to the anisotropic interaction with the CNT(15,0) surface; (2*J* + 1) of the m-sublevels are equally populated for each *J* in the spherical harmonics *Y*_*Jm*_(*χ*, *ω*) without any external interaction^3,21,58^. Breaking of the degeneracy lets each shell-type species have the preferred orientation upon the adsorption, inducing the hindered rotation around the potential energy minimum^59–61^. Actually, the all of ortho, para-2, and ortho-2 tend to form edge-on attaching to the CNT(15,0) surface.(Supplementary Fig. 10).

### Equations of motion

The EOMs to time-evolve **R**_COM_(*t*), *R*_rel_(*t*), Ω_A_(*t*), **P**_COM_(*t*), *P*_rel_(*t*), and Π_A_(*t*) specifying dynamics of both the NWPs and EWPs can be derived through the time-dependent quantum variational principle that minimizes the action integral for the time-dependent nuclear-electron wave function ${{{\Psi }}}_{{{{{{{{\rm{AB}}}}}}}},J}^{{{{{{{{\rm{L,S}}}}}}}}}(t)$ defined as

7 $${{\Gamma }}=\int\,dt \left\langle {{{\Psi }}}_{{{{{{{{\rm{AB}}}}}}}},J}^{{{{{{{{\rm{L,S}}}}}}}}}(t)\left| i\frac{\partial }{\partial t}-\hat{H}\right| {{{\Psi }}}_{{{{{{{{\rm{AB}}}}}}}},J}^{{{{{{{{\rm{L,S}}}}}}}}}(t)\right\rangle ,$$

with the Hamiltonian operator $\hat{H}$. (Supplementary Sections 1C and 1E) The time-dependent variational principle^62^, *δ*Γ/*δ***R**_COM_(*t*) = 0, etc., yields the EOMs,

8 $${\dot{{{{{{{{\bf{P}}}}}}}}}}_{{{{{{{{\rm{COM}}}}}}}}}(t)=-\frac{1}{2}\frac{\partial {E}_{{{{{{{{\rm{tot}}}}}}}},J}}{\partial {{{{{{{{\bf{R}}}}}}}}}_{{{{{{{{\rm{COM}}}}}}}}}(t)}-\frac{1}{2}\frac{\partial {E}_{{{{{{{{\rm{CNT}}}}}}}},J}(\frac{{D}_{{{{{{{{\rm{CNT}}}}}}}}}}{2}-r,\theta )}{\partial {{{{{{{{\bf{R}}}}}}}}}_{{{{{{{{\rm{COM}}}}}}}}}(t)},$$

9 $${\dot{{{{{{{{\bf{P}}}}}}}}}}_{{{{{{{{\rm{rel}}}}}}}}}(t)=-2\frac{\partial {E}_{{{{{{{{\rm{tot}}}}}}}},J}}{\partial {R}_{{{{{{{{\rm{rel}}}}}}}}}(t)}\frac{{{{{{{{{\bf{R}}}}}}}}}_{{{{{{{{\rm{rel}}}}}}}}}(t)}{{R}_{{{{{{{{\rm{rel}}}}}}}}}(t)}-2\frac{\partial {E}_{{{{{{{{\rm{CNT}}}}}}}},J}(\frac{{D}_{{{{{{{{\rm{CNT}}}}}}}}}}{2}-r,\theta )}{\partial {{{{{{{{\bf{R}}}}}}}}}_{{{{{{{{\rm{rel}}}}}}}}}(t)},$$

10 $${\dot{{{\Pi }}}}_{{{{{{{{\rm{A}}}}}}}}}(t)=-\frac{1}{4}\frac{\partial {E}_{{{{{{{{\rm{tot}}}}}}}},J}}{\partial {{{\Omega }}}_{{{{{{{{\rm{A}}}}}}}}}(t)},$$

11 $${\dot{{{{{{{{\bf{R}}}}}}}}}}_{{{{{{{{\rm{COM}}}}}}}}}(t)=\frac{{{{{{{{{\bf{P}}}}}}}}}_{{{{{{{{\rm{COM}}}}}}}}}(t)}{M},$$

12 $${\dot{{{{{{{{\bf{R}}}}}}}}}}_{{{{{{{{\rm{rel}}}}}}}}}(t)=\frac{{{{{{{{{\bf{P}}}}}}}}}_{{{{{{{{\rm{rel}}}}}}}}}(t)}{M},$$

and

13 $${\dot{{{\Omega }}}}_{{{{{{{{\rm{A}}}}}}}}}(t)=\frac{{{{\Pi }}}_{{{{{{{{\rm{A}}}}}}}}}(t)}{M}.$$

We note that the anisotropic interaction with the CNT(15,0) surface breaks the spatial translational symmetry on the cross section of CNT(15,0), enabling the introduction of a laboratory coordinate system. Time evolution of the EWPs can be fully specified by the dynamics of the NWPs based on the assumptions that the EWP dynamics is much faster than the NWP dynamics and that the EWPs instantly adjust their widths and center positions to the NWP dynamics at each moment, which is the reason why momenta of the EWP center position and width are removed^41–49^. (Supplementary Section 1A) It is remarkable that the above EOMs are universal and can be systematically applicable not only to the current four shell-type species but also to shell-type species possessing higher-energy nuclear quantum rotation.

### Simulation details

The explicit and analytical derivation of the simple EOMs (8)–(13) makes it possible to calculate real-time microscopic trajectories of hydrogen molecules possessing the different nuclear quantum rotation in the collision dynamics with the SWCNT. All the integrations of the EOMs were performed by the velocity-verlet method with the time step 0.1 fs in the NVE (microcanonical) simulations. We started all the collision dynamics up to 500 ps from **R**_COM_(0) = (0.00000 Å, 2.645885 Å, 0.00000 Å) with the same initial velocity **P**_COM_(0) = (0.00000 Å *fs*^−1^, −1.85952 × 10^−2^ Å *fs*^−1^, 0.00000 Å *fs*^−1^), **P**_rel_(0) = (4.37534 × 10^−3^ Å *fs*^−1^, 0.00000 Å *fs*^−1^, 0.00000 Å *fs*^−1^), and Π_A_(0) = (0.00000 Å *fs*^−1^, 0.00000 Å *fs*^−1^, 0.00000 Å *fs*^−1^) but with the different initial angles *θ*(0) between the molecular axis and the CNT(15,0) surface set as 0 degree, 45 degree, and 90 degree which will be called CNT-0, CNT-45, and CNT-90 in this Article, respectively. See more details in Supplementary Section 1.

### Optimized molecular structure

In Fig. 2a, we freely optimized ${{{{{{{{\bf{R}}}}}}}}}_{{{{{{{{\rm{e}}}}}}}}}^{{{{{{{{\rm{L}}}}}}}}}(t)$, ${{{{{{{{\bf{R}}}}}}}}}_{{{{{{{{\rm{e}}}}}}}}}^{{{{{{{{\rm{S}}}}}}}}}(t)$, *ρ*_L_(*t*), and *ρ*_S_(*t*) of the EWPs and Ω_A_(*t*) of the NWPs to find the minimum of the total molecular energy *E*_tot,*J*_ along *R*_rel_(*t*) with **R**_COM_(*t*) = (0, 0, 0) and all the momenta set to be zero. The hydrogen molecular structure is solely specified with the NWP center position and width (Fig. 2d, Supplementary Fig. 1, Supplementary Fig. 2a and Supplementary Table 2) as well as the EWP width and center position (Supplementary Fig. 3, Supplementary Fig. 4, Supplementary Fig. 2b, c and Supplementary Table 3). Both of the NWP and EWPs at the energy minima are more delocalized with the higher rotational excitation, which stems from the stronger centrifugal force and is directly linked to the elongation of the H-H bond length in the higher rotational state. Figure 2d and Supplementary Fig. 2a demonstrate the relationship *R*_rel_(*t*) ≫ Ω_A_(*t*) around the stable H-H bond length regardless of the rotational states, validating $\exp [-{R}_{{{{{{{{\rm{rel}}}}}}}}}^{2}(t)/4{{{\Omega }}}_{{{{{{{{\rm{A}}}}}}}}}^{2}(t)]\ll 1$ and thus its neglection in the derivation of the EOMs (8)–(13). The EWPs are always located at the molecular center being independent of the H-H bond length. (Supplementary Fig. 3) Similar to Gaussian obtained by the G-NEWPMD method, the hydrogen molecule is composed of simple two Gaussian EWPs of the different width. (Supplementary Table 3) The change of the EWPs in Supplementary Fig. 2b, c is purely caused by the nuclear rotational excitation, meaning that an electronic wave function is modified according to a nuclear wave function even on the same electronic state, which should be distinguished from the conventional insight that a nuclear wave function is modified according to an electronic wave function on an electronically excited state. In other words, the NEWPMD-QR method proposes that the same electronic potential function should not be used on a different nuclear excited state^2,5,39^. The width of the NWPs and EWPs obtained by the G-NEWPMD method (Supplementary Tables 2 and 3) is larger than any corresponding width calculated by the NEWPMD-QR method, which is related to the longer H-H bond length in the former method than in the latter method; the two distinct Gaussian NWPs in the G-NEWPMD method induce a stronger repulsion force between the two nuclei than the shell-type NWPs in the NEWPMD-QR method^41–49^.

## Supplementary information

Deuk_PR File

Supplementary Information

Description of Additional Supplementary Files

Supplementary Movie 1

Supplementary Movie 2

Supplementary Movie 3

Supplementary Movie 4

Supplementary Movie 5

## Data Availability

Data sharing not applicable to this article as no datasets were generated or analysed during the current study.
