# Supplementary material for: Real-time hydrogen molecular dynamics satisfying the nuclear spin statistics of a quantum rotor
Source: Commun Chem. 2022 Dec 3;5:168. doi: 10.1038/s42004-022-00788-z (PMC9814564; doi:10.1038/s42004-022-00788-z)
Supplement: Supplementary file 2 — Supplementary Information [file 42004_2022_788_MOESM2_ESM.pdf]

Real-Time Hydrogen Molecular Dynamics Satisfying the Nuclear Spin Statistics of a Quantum Rotor

Kim Hyeon-Deuk* and I-Ya Chang

Department of Chemistry, Kyoto University, Kyoto, 606-8502, Japan

E-mail: kim@kuchem.kyoto-u.ac.jp

*To whom correspondence should be addressed

Supplementary Methods

S1. Computational methods: the nuclear and electron wave packet molecular dynamics method with quantum rotation

We developed the non-empirical quantum molecular dynamics method satisfying the nuclear spin statistics of a quantum rotor called the nuclear and electron wave packet molecular dynamics method with quantum rotation (the NEWPMD-QR method) by extending the original NEWPMD method free from the nuclear spin statistics and will be called the Gaussian NEWPMD method (the G-NEWPMD method) in this Article. As will be explained below, the NEWPMD-QR method provides equations of motion (EOMs) to time-evolve a nuclear-electron wave function of a rotationally-excited hydrogen molecule.

S1A. Time-dependent nuclear-electron wave function satisfying the nuclear spin statistics of a quantum rotor

The NEWPMD-QR method describes two nuclei, A and B, by nuclear wave packets (NWPs) via the time-dependent Hartree approach, while it expresses two electrons, L and S, by two Gaussian electron wave packets (EWPs) through the perfect-pairing (PP) valence bond (VB) theory that appropriately treats the Pauli exclusion energy. The time-dependent nuclear-electron wave function for a hydrogen molecule composed of the two nuclei A and B and two electrons L and S with the rotational quantum number J is introduced as

$$\Psi^{\mathrm{L,S}}_{\mathrm{AB},J}(t) \quad = \quad \Phi_{\mathrm{AB},J}(\mathbf{Q}_1, \mathbf{Q}_2, t)\psi_{\mathrm{L,S}}(\mathbf{q}_1, \mathbf{q}_2, t), \tag{1}$$

where $\mathbf{Q}_1$, $\mathbf{Q}_2$, $\mathbf{q}_1$ and $\mathbf{q}_2$ denote A-, B-, L- and S-coordinates, respectively. The normalized time-dependent PP VB electronic wave function for the two electrons is expressed as

$$\begin{aligned}\psi_{\mathrm{L,S}}(\mathbf{q}_1,\mathbf{q}_2,t) &= \frac{1}{2^{\frac{1}{2}}(1+S_{\mathrm{LS}}^2(t))^{\frac{1}{2}}}(\phi_{\mathrm{L}}(\mathbf{q}_1,t)\phi_{\mathrm{S}}(\mathbf{q}_2,t)+\phi_{\mathrm{S}}(\mathbf{q}_1,t)\phi_{\mathrm{L}}(\mathbf{q}_2,t)) \\ &\quad \times\frac{1}{2^{\frac{1}{2}}}(\alpha(1)\beta(2)-\beta(2)\alpha(1)). \end{aligned} \tag{2}$$

Here, α and β are electron spin functions with the two electrons forming the singlet. The normalized Gaussian EWP $\phi_k(\mathbf{q},t)$ is specified by the position of the EWP center $\mathbf{R}_{\mathrm{e}}^k(t) = (X_{\mathrm{e}}^k(t), Y_{\mathrm{e}}^k(t), Z_{\mathrm{e}}^k(t))$ and the EWP width $\rho_k(t)$:

$$\phi_k(\mathbf{q},t) = \frac{1}{N_k}\exp\left[-\frac{(\mathbf{q}-\mathbf{R}_{\mathrm{e}}^k(t))^2}{4\rho_k^2(t)}\right], \tag{3}$$

with the normalization factor $N_k = (2\pi\rho_k^2)^{3/4}$ for k =L and S. For simplicity, $\hbar = 1$ and the electron charge and mass are set as unity for scaling. $S_{\mathrm{LS}}(t)$ is the overlap integral between ϕ_{L} and ϕ_{S}:

$$S_{\mathrm{LS}}(t) = \int \mathrm{d}\mathbf{q}_1\phi_{\mathrm{L}}^*(\mathbf{q}_1,t)\phi_{\mathrm{S}}(\mathbf{q}_1,t) \tag{4}$$

$$= \left(\frac{2\rho_{\mathrm{L}}(t)\rho_{\mathrm{S}}(t)}{\rho_{\mathrm{L}}^2(t)+\rho_{\mathrm{S}}^2(t)}\right)^{\frac{3}{2}}\exp\left[-\frac{|\mathbf{R}_{\mathrm{e}}^{\mathrm{L}}(t)-\mathbf{R}_{\mathrm{e}}^{\mathrm{S}}(t)|^2}{4(\rho_{\mathrm{L}}^2(t)+\rho_{\mathrm{S}}^2(t))}\right]. \tag{5}$$

In order to obtain the time-dependent nuclear wave function for the two hydrogen nuclei A and B with the rotational quantum number J, $\Phi_{\mathrm{AB},J}(\mathbf{Q}_1,\mathbf{Q}_2,t)$, we start with a normalized Gaussian NWP specified by the NWP center position $\mathbf{R}_k(t)$ and width $\Omega_k(t)$ accompanied with their conjugate momenta $\mathbf{P}_k(t)$ and $\Pi_k(t)$, respectively, as

$$\begin{aligned}\tilde{\Phi}_k(\mathbf{Q},t) &= \left(\frac{1}{2\pi\Omega_k^2(t)}\right)^{\frac{3}{4}}\times \\ &\quad \exp\left[-\frac{(\mathbf{Q}-\mathbf{R}_k(t))^2}{4\Omega_k^2(t)}+\frac{\mathrm{i}\Pi_k(t)(\mathbf{Q}-\mathbf{R}_k(t))^2}{2\Omega_k(t)}+\mathrm{i}\mathbf{P}_k(t)\cdot(\mathbf{Q}-\mathbf{R}_k(t))\right],\end{aligned} \tag{6}$$

for K =A or B. The time-dependent nuclear-electron wave function for a hydrogen molecule introduced in the G-NEWPMD method can be written by simply adopting the Gaussian NWPs and Gaussian EWPs as

$$\Psi^{\mathrm{L,S}}_{\mathrm{A,B}}(t) = \tilde{\Phi}_{\mathrm{A}}(\mathbf{Q}_1, t)\tilde{\Phi}_{\mathrm{B}}(\mathbf{Q}_2, t)\psi_{\mathrm{L,S}}(\mathbf{q}_1, \mathbf{q}_2, t). \tag{7}$$

Figure 1(a) shows the nuclear distribution of the stable hydrogen molecule, $\tilde{\Phi}^2_{\mathrm{A}}(\mathbf{Q}, t)\tilde{\Phi}^2_{\mathrm{B}}(\mathbf{Q}, t)$, calculated by the G-NEWPMD method. Its nuclear distribution has the dumbbell-shape nuclear delocalization composed of the two distinct Gaussian NWPs. Therefore, we will call this hydrogen molecule calculated by the G-NEWPMD method Gaussian throughout this Article.

The Hartree product of the two normalized Gaussian NWPs,

$$\begin{aligned}
&\tilde{\Phi}_{\mathrm{A}}(\mathbf{Q}_1, t)\tilde{\Phi}_{\mathrm{B}}(\mathbf{Q}_2, t) = \left(\frac{1}{2\pi\Omega^2_{\mathrm{A}}(t)}\right)^{\frac{3}{4}} \left(\frac{1}{2\pi\Omega^2_{\mathrm{B}}(t)}\right)^{\frac{3}{4}} \times \\
&\exp\left[-\frac{(\mathbf{Q}_1 - \mathbf{R}_{\mathrm{A}}(t))^2}{4\Omega^2_{\mathrm{A}}(t)} + \frac{\mathrm{i}\Pi_{\mathrm{A}}(t)(\mathbf{Q}_1 - \mathbf{R}_{\mathrm{A}}(t))^2}{\Omega_{\mathrm{A}}(t)} + \mathrm{i}\mathbf{P}_{\mathrm{A}}(t)\cdot(\mathbf{Q}_1 - \mathbf{R}_{\mathrm{A}}(t))\right] \\
&\exp\left[-\frac{(\mathbf{Q}_2 - \mathbf{R}_{\mathrm{B}}(t))^2}{4\Omega^2_{\mathrm{B}}(t)} + \frac{\mathrm{i}\Pi_{\mathrm{B}}(t)(\mathbf{Q}_2 - \mathbf{R}_{\mathrm{B}}(t))^2}{2\Omega_{\mathrm{B}}(t)} + \mathrm{i}\mathbf{P}_{\mathrm{B}}(t)\cdot(\mathbf{Q}_2 - \mathbf{R}_{\mathrm{B}}(t))\right],
\end{aligned} \tag{8}$$

can be transformed with the center-of-mass (COM) coordinates

$$\mathbf{Q}_{\mathrm{COM}} = \frac{\mathbf{Q}_1 + \mathbf{Q}_2}{2}, \tag{9}$$

and the relative coordinates

$$\mathbf{Q}_{\mathrm{rel}} = \mathbf{Q}_1 - \mathbf{Q}_2, \tag{10}$$

to

$$\tilde{\Phi}_{\mathrm{A}}(\mathbf{Q}_1, t)\tilde{\Phi}_{\mathrm{B}}(\mathbf{Q}_2, t) = \left(\frac{1}{2\pi\Omega^2_{\mathrm{A}}(t)}\right)^{\frac{3}{4}} \left(\frac{1}{2\pi\Omega^2_{\mathrm{B}}(t)}\right)^{\frac{3}{4}} \times \tag{11}$$

$$
\exp\left[-\frac{(\mathbf{Q}_{\mathrm{COM}}+\mathbf{Q}_{\mathrm{rel}}/2-\mathbf{R}_{\mathrm{A}}(t))^2}{4\Omega_{\mathrm{A}}^2(t)}+\frac{\mathrm{i}\Pi_{\mathrm{A}}(t)(\mathbf{Q}_{\mathrm{COM}}+\mathbf{Q}_{\mathrm{rel}}/2-\mathbf{R}_{\mathrm{A}}(t))^2}{2\Omega_{\mathrm{A}}(t)}\right.
$$
$$
\left.+\mathrm{i}\mathbf{P}_{\mathrm{A}}(t)\cdot(\mathbf{Q}_{\mathrm{COM}}+\mathbf{Q}_{\mathrm{rel}}/2-\mathbf{R}_{\mathrm{A}}(t))\right]
$$
$$
\exp\left[-\frac{(\mathbf{Q}_{\mathrm{COM}}-\mathbf{Q}_{\mathrm{rel}}/2-\mathbf{R}_{\mathrm{B}}(t))^2}{4\Omega_{\mathrm{B}}^2(t)}+\frac{\mathrm{i}\Pi_{\mathrm{B}}(t)(\mathbf{Q}_{\mathrm{COM}}-\mathbf{Q}_{\mathrm{rel}}/2-\mathbf{R}_{\mathrm{B}}(t))^2}{2\Omega_{\mathrm{B}}(t)}\right.
$$
$$
\left.+\mathrm{i}\mathbf{P}_{\mathrm{B}}(t)\cdot(\mathbf{Q}_{\mathrm{COM}}-\mathbf{Q}_{\mathrm{rel}}/2-\mathbf{R}_{\mathrm{B}}(t))\right]
$$
$$
= \left(\frac{1}{2\pi\Omega_{\mathrm{A}}^2(t)}\right)^{\frac{3}{2}}\exp\left[-\frac{(\mathbf{Q}_{\mathrm{COM}}-\mathbf{R}_{\mathrm{COM}}(t))^2}{2\Omega_{\mathrm{A}}^2(t)}-\frac{(\mathbf{Q}_{\mathrm{rel}}-\mathbf{R}_{\mathrm{rel}}(t))^2}{8\Omega_{\mathrm{A}}^2(t)}\right. \tag{12}
$$
$$
+\mathrm{i}\frac{\Pi_{\mathrm{A}}(t)}{\Omega_{\mathrm{A}}(t)}(\mathbf{Q}_{\mathrm{COM}}-\mathbf{R}_{\mathrm{COM}}(t))^2+\mathrm{i}\frac{\Pi_{\mathrm{A}}(t)}{4\Omega_{\mathrm{A}}(t)}(\mathbf{Q}_{\mathrm{rel}}-\mathbf{R}_{\mathrm{rel}}(t))^2
$$
$$
\left.+2\mathrm{i}\mathbf{P}_{\mathrm{COM}}(t)\cdot(\mathbf{Q}_{\mathrm{COM}}-\mathbf{R}_{\mathrm{COM}}(t))+\mathrm{i}\frac{\mathbf{P}_{\mathrm{rel}}(t)}{2}\cdot(\mathbf{Q}_{\mathrm{rel}}-\mathbf{R}_{\mathrm{rel}}(t))\right].
$$

Here, we defined the COM position as $\mathbf{R}_{\mathrm{COM}}(t)=(\mathbf{R}_{\mathrm{A}}(t)+\mathbf{R}_{\mathrm{B}}(t))/2$ and the relative position defined by the distance between the two NWP center positions as $\mathbf{R}_{\mathrm{rel}}(t)=\mathbf{R}_{\mathrm{A}}(t)-\mathbf{R}_{\mathrm{B}}(t)$ and their conjugate momenta as $\mathbf{P}_{\mathrm{COM}}(t)$ and $\mathbf{P}_{\mathrm{rel}}(t)$, respectively. The three dimensional absolute coordinates $\mathbf{Q}_1$ and $\mathbf{Q}_2$ were transformed to the COM and relative coordinates $\mathbf{Q}_{\mathrm{COM}}$ and $\mathbf{Q}_{\mathrm{rel}}$ to effectively express the nuclear quantum rotation.

We calculate hydrogen molecules in the ground ($J=0$), first-excited ($J=1$), second-excited ($J=2$) and third-excited ($J=3$) quantum rotational states that will be called para, ortho, para-2, and ortho-2 throughout this Article, respectively. Although para on the ground rotational state should have a completely spherical nuclear wave function, eq.(12) itself is a simple deformation of the Hartree product of the paired Gaussian NWPs, $\tilde{\Phi}_{\mathrm{A}}(\mathbf{Q}_1,t)\tilde{\Phi}_{\mathrm{B}}(\mathbf{Q}_2,t)$, and cannot describe such spherical nuclear wave function. Therefore, to obtain the time-dependent nuclear wave function with the nuclear quantum rotation, we modify eq.(12) as

$$
\tilde{\Phi}_{\mathrm{A}}(\mathbf{Q}_1,t)\tilde{\Phi}_{\mathrm{B}}(\mathbf{Q}_2,t)\simeq\exp\left[-\frac{(\mathbf{Q}_{\mathrm{COM}}-\mathbf{R}_{\mathrm{COM}}(t))^2}{2\Omega_{\mathrm{A}}^2(t)}-\frac{(Q_{\mathrm{rel}}-R_{\mathrm{rel}}(t))^2}{8\Omega_{\mathrm{A}}^2(t)}\right. \tag{13}
$$
$$
+\mathrm{i}\frac{\Pi_{\mathrm{A}}(t)}{\Omega_{\mathrm{A}}(t)}(\mathbf{Q}_{\mathrm{COM}}-\mathbf{R}_{\mathrm{COM}}(t))^2+\mathrm{i}\frac{\Pi_{\mathrm{A}}(t)}{4\Omega_{\mathrm{A}}(t)}(Q_{\mathrm{rel}}-R_{\mathrm{rel}}(t))^2
$$
$$
\left.+2\mathrm{i}\mathbf{P}_{\mathrm{COM}}(t)\cdot(\mathbf{Q}_{\mathrm{COM}}-\mathbf{R}_{\mathrm{COM}}(t))+\mathrm{i}\frac{P_{\mathrm{rel}}(t)}{2}(Q_{\mathrm{rel}}-R_{\mathrm{rel}}(t))\right],
$$

where $Q_{\rm rel} = |\mathbf{Q}_{\rm rel}|$, $R_{\rm rel}(t) = |\mathbf{R}_{\rm A}(t) - \mathbf{R}_{\rm B}(t)|$ and $P_{\rm rel}(t) = |\mathbf{P}_{\rm rel}(t)|$. Now eq.(13) can express the completely spherical shell-shape nuclear wave function for para owing to the spherical rotational wave function, $\exp[-(Q_{\rm rel} - R_{\rm rel}(t))^2/8\Omega_{\rm A}^2(t)]$. Since the total nuclear wave function must be symmetric and antisymmetric with the anti-parallel and parallel nuclear spins, respectively, we further multiply the spherical harmonics, $Y_{Jm}(\chi,\omega)$, to eq.(13) as the angular wave function for para ($J = 0$), ortho ($J = 1$), para-2 ($J = 2$), and ortho-2 ($J = 3$), finally obtaining

$$\begin{aligned}\Phi_{{\rm AB},J}(\mathbf{Q}_1,\mathbf{Q}_2,t) &= N_{{\rm AB},J}^{-1}\exp\left[-\frac{(\mathbf{Q}_{\rm COM}-\mathbf{R}_{\rm COM}(t))^2}{2\Omega_{\rm A}^2(t)} - \frac{(Q_{\rm rel}-R_{\rm rel}(t))^2}{8\Omega_{\rm A}^2(t)}\right.\\ &\quad +{\rm i}\frac{\Pi_{\rm A}(t)}{\Omega_{\rm A}(t)}(\mathbf{Q}_{\rm COM}-\mathbf{R}_{\rm COM}(t))^2 + {\rm i}\frac{\Pi_{\rm A}(t)}{4\Omega_{\rm A}(t)}(Q_{\rm rel}-R_{\rm rel}(t))^2\\ &\quad \left. +2{\rm i}\mathbf{P}_{\rm COM}(t)\cdot(\mathbf{Q}_{\rm COM}-\mathbf{R}_{\rm COM}(t)) + {\rm i}\frac{P_{\rm rel}(t)}{2}(Q_{\rm rel}-R_{\rm rel}(t))\right] Y_{Jm}(\chi,\omega).\end{aligned} \tag{14}$$

It should be noted that χ and ω in $Y_{Jm}(\chi,\omega)$ do not specify the actual H-H molecular axis but denote the angular coordinates of $\mathbf{Q}_{\rm rel}$ from the x-axis and from the y-axis on the yz-plane, respectively, that is,

$$Q_{{\rm rel},x} = Q_{\rm rel}\cos\chi, \quad Q_{{\rm rel},y} = Q_{\rm rel}\sin\chi\cos\omega, \quad Q_{{\rm rel},z} = Q_{\rm rel}\sin\chi\sin\omega. \tag{15}$$

Note that, in the NEWPMD-QR method, the normalization factor $N_{{\rm AB},J}$ depends on the rotational quantum number J:

$$\begin{aligned}&N_{{\rm AB},J} = C_J\pi^{\frac{5}{4}}\Omega_{\rm A}^2 \times\\ &\left\{2\exp\left[-\frac{R_{\rm rel}^2(t)}{4\Omega_{\rm A}^2(t)}\right]R_{\rm rel}\Omega_{\rm A} + \pi^{\frac{1}{2}}\left(R_{\rm rel}^2(t)+2\Omega_{\rm A}^2(t)\right)\left(1+{\rm erf}\left[\frac{R_{\rm rel}(t)}{2\Omega_{\rm A}(t)}\right]\right)\right\}^{\frac{1}{2}},\end{aligned} \tag{16}$$

with $C_0 = 2$ for para, $C_1 = 2/\sqrt{3}$ for ortho, $C_2 = 4/\sqrt{5}$ for para-2, and $C_3 = 4/\sqrt{7}$ for ortho-2. Figure 1(b-f) display the most stable nuclear distributions $\Phi_{{\rm AB},J}^*(\mathbf{Q},\mathbf{Q},t)\Phi_{{\rm AB},J}(\mathbf{Q},\mathbf{Q},t)$ of para, ortho, para-2, and ortho-2 calculated by the NEWPMD-QR method.

S1B. Kinetic energy and electrostatic interaction energy

The total molecular energy, $E_{\mathrm{tot},J}$, the sum of the kinetic energy of the hydrogen nuclei and electrons as well as the three electrostatic interaction energy of electron-electron, nucleus-nucleus and nucleus-electron,

$$E_{\mathrm{tot},J} \equiv E_{\mathrm{ke,elec}} + E_{\mathrm{ee}} + E_{\mathrm{ke,nuc},J} + E_{\mathrm{nn}} + E_{\mathrm{ne}}, \tag{17}$$

is indispensable for deriving the EOMs. Because the electronic wave function for the two electrons, eq.(2), is common in the NEWPMD-QR and G-NEWPMD methods, the kinetic energy of the two electrons

$$\begin{aligned} E_{\mathrm{ke,elec}} &= \langle \Psi^{\mathrm{L,S}}_{\mathrm{AB},J}(t)| -\frac{1}{2}\frac{\partial^2}{\partial \mathbf{q}_1^2} - \frac{1}{2}\frac{\partial^2}{\partial \mathbf{q}_2^2} |\Psi^{\mathrm{L,S}}_{\mathrm{AB},J}(t)\rangle & (18)\\ &= \frac{T_{LL}(t) + T_{SS}(t) + 2S_{\mathrm{LS}}(t)T_{\mathrm{LS}}(t)}{1 + S^2_{\mathrm{LS}}(t)}, & (19)\end{aligned}$$

with the one-electron kinetic energy

$$T_{ij}(t) = \frac{1}{4(\rho_i^2(t) + \rho_j^2(t))}\left(3 - \frac{|\mathbf{R}^i_{\mathrm{e}}(t) - \mathbf{R}^j_{\mathrm{e}}(t)|^2}{2(\rho_i^2(t) + \rho_j^2(t))}\right) S_{ij}(t), \tag{20}$$

and the electron-electron interaction energy

$$\begin{aligned} E_{\mathrm{ee}} &= \langle \Psi^{\mathrm{L,S}}_{\mathrm{AB},J}(t)| \frac{1}{|\mathbf{q}_1 - \mathbf{q}_2|} |\Psi^{\mathrm{L,S}}_{\mathrm{AB},J}(t)\rangle & (21)\\ &= \frac{(LL|SS) + (LS|SL)}{1 + S^2_{\mathrm{LS}}}, & (22)\end{aligned}$$

with the two-electron integral

$$\begin{aligned} (ij|kl) &= \int\int \mathrm{d}\mathbf{q}_1 \mathrm{d}\mathbf{q}_2 \phi_i^*(\mathbf{q}_1)\phi_j(\mathbf{q}_1)\frac{1}{|\mathbf{q}_1 - \mathbf{q}_2|}\phi_k^*(\mathbf{q}_2)\phi_l(\mathbf{q}_2) & (23)\\ &= \pi^3 N_i N_j N_k N_l \, (\alpha+\beta)^{-3/2} \, (\gamma+\delta)^{-3/2} \, |\mathbf{r}_{\mathrm{p}} - \mathbf{r}_{\mathrm{q}}|^{-1} \end{aligned}$$

$$\exp[-\alpha\beta(\alpha+\beta)^{-1}|\mathbf{r}_i-\mathbf{r}_j|^2-\gamma\delta(\gamma+\delta)^{-1}|\mathbf{r}_k-\mathbf{r}_l|^2]$$
$$\mathrm{erf}[(\alpha+\beta)^{1/2}(\gamma+\delta)^{1/2}(\alpha+\beta+\gamma+\delta)^{-1/2}|\mathbf{r}_\mathrm{p}-\mathbf{r}_\mathrm{q}|] \qquad (24)$$

where $\alpha = 1/4\rho_i^2$, $\beta = 1/4\rho_j^2$, $\gamma = 1/4\rho_k^2$, and $\delta = 1/4\rho_l^2$, and

$$\mathbf{r}_\mathrm{p} = \frac{\alpha\mathbf{r}_i+\beta\mathbf{r}_j}{\alpha+\beta}, \quad \mathbf{r}_\mathrm{q} = \frac{\gamma\mathbf{r}_k+\delta\mathbf{r}_l}{\gamma+\delta}, \qquad (25)$$

are the same both in the NEWPMD-QR and G-NEWPMD methods. Note that $\langle\Psi^{\mathrm{L,S}}_{\mathrm{AB},J}(t)|\cdots|\Psi^{\mathrm{L,S}}_{\mathrm{AB},J}(t)\rangle$ means a quantum expectation with the nuclear-electron wave function $\Psi^{\mathrm{L,S}}_{\mathrm{AB},J}(t)$.

On the contrary, the kinetic energy of the hydrogen nuclei and the electrostatic interaction energy of nucleus-nucleus and nucleus-electron significantly differ between the NEWPMD-QR and G-NEWPMD methods. The kinetic energy of the hydrogen nuclei depending on the rotational quantum number J is derived in the NEWPMD-QR method as

$$E_{\mathrm{ke,nuc},J} = \langle\Psi^{\mathrm{L,S}}_{\mathrm{AB},J}(t)| -\frac{1}{2M_\mathrm{nuc}}\frac{\partial^2}{\partial\mathbf{Q}_1^2}-\frac{1}{2M_\mathrm{nuc}}\frac{\partial^2}{\partial\mathbf{Q}_2^2}|\Psi^{\mathrm{L,S}}_{\mathrm{AB},J}(t)\rangle \qquad (26)$$
$$= \langle\Psi^{\mathrm{L,S}}_{\mathrm{AB},J}(t)| -\frac{1}{4M_\mathrm{nuc}}\frac{\partial^2}{\partial\mathbf{Q}_\mathrm{COM}^2}-\frac{1}{M_\mathrm{nuc}}\frac{\partial^2}{\partial Q_\mathrm{rel}^2}-\frac{2}{M_\mathrm{nuc}Q_\mathrm{rel}}\frac{\partial}{\partial Q_\mathrm{rel}} \qquad (27)$$
$$-\frac{1}{M_\mathrm{nuc}Q_\mathrm{rel}^2}\frac{\partial^2}{\partial\chi^2}-\frac{1}{M_\mathrm{nuc}Q_\mathrm{rel}^2}\cot\chi\frac{\partial}{\partial\chi}|\Psi^{\mathrm{L,S}}_{\mathrm{AB},J}(t)\rangle$$
$$= \frac{\mathbf{P}^2_\mathrm{COM}(t)}{M_\mathrm{nuc}}+\frac{P^2_\mathrm{rel}(t)}{4M_\mathrm{nuc}} \qquad (28)$$
$$+\frac{2R_\mathrm{rel}(t)\Omega_\mathrm{A}(t)P_\mathrm{rel}(t)\Pi_\mathrm{A}(t)}{M_\mathrm{nuc}(R^2_\mathrm{rel}(t)+2\Omega^2_\mathrm{A}(t))}+\frac{2\Pi^2_\mathrm{A}(t)(R^2_\mathrm{rel}(t)+3\Omega^2_\mathrm{A}(t))}{M_\mathrm{nuc}(R^2_\mathrm{rel}(t)+2\Omega^2_\mathrm{A}(t))}+\frac{R^2_\mathrm{rel}(t)+(2J(J+1)+3)\Omega^2_\mathrm{A}(t)}{2M_\mathrm{nuc}\Omega^2_\mathrm{A}(t)(R^2_\mathrm{rel}(t)+2\Omega^2_\mathrm{A}(t))}.$$

M_nuc is relative mass of the hydrogen nucleus to the electron.

Interestingly, the electrostatic interaction energy of nucleus-nucleus calculated by the NEWPMD-QR method was found to be independent of the rotational quantum number J,

$$E_\mathrm{nn} = \langle\Psi^{\mathrm{L,S}}_{\mathrm{AB},J}(t)|\frac{1}{|\mathbf{Q}_1-\mathbf{Q}_2|}|\Psi^{\mathrm{L,S}}_{\mathrm{AB},J}(t)\rangle \qquad (29)$$
$$= \langle\Psi^{\mathrm{L,S}}_{\mathrm{AB},J}(t)|\frac{1}{Q_\mathrm{rel}}|\Psi^{\mathrm{L,S}}_{\mathrm{AB},J}(t)\rangle \qquad (30)$$

$$= \frac{2\exp\left[-\frac{R_{\rm rel}^2(t)}{4\Omega_{\rm A}^2(t)}\right]\Omega_{\rm A}(t)+\sqrt{\pi}R_{\rm rel}(t)\left(1+{\rm erf}\left[\frac{R_{\rm rel}(t)}{2\Omega_{\rm A}(t)}\right]\right)}{2\exp\left[-\frac{R_{\rm rel}^2(t)}{4\Omega_{\rm A}^2(t)}\right]\Omega_{\rm A}(t)R_{\rm rel}(t)+\sqrt{\pi}(R_{\rm rel}^2(t)+2\Omega_{\rm A}^2(t))\left(1+{\rm erf}\left[\frac{R_{\rm rel}(t)}{2\Omega_{\rm A}(t)}\right]\right)}. \quad (31)$$

This is because the radial nucleus-nucleus interaction depends only on the internuclear distance $Q_{\rm rel}$, not being influenced by the angular coordinates χ and ω.

The electrostatic interaction energy of nucleus-electron is also independent of the rotational quantum number J due to their radial Coulomb interaction free from the angular coordinates, being derived as

$$E_{\rm ne} = -\langle\Psi^{\rm L,S}_{{\rm AB},J}(t)|\frac{1}{|\mathbf{q}_1-\mathbf{Q}_1|}+\frac{1}{|\mathbf{q}_2-\mathbf{Q}_1|}+\frac{1}{|\mathbf{q}_1-\mathbf{Q}_2|}+\frac{1}{|\mathbf{q}_2-\mathbf{Q}_2|}|\Psi^{\rm L,S}_{{\rm AB},J}(t)\rangle \quad (32)$$

$$= -\langle\Psi^{\rm L,S}_{{\rm AB},J}(t)|\frac{1}{|\mathbf{q}_1-\mathbf{Q}_{\rm COM}-\frac{\mathbf{Q}_{\rm rel}}{2}|}+\frac{1}{|\mathbf{q}_2-\mathbf{Q}_{\rm COM}-\frac{\mathbf{Q}_{\rm rel}}{2}|} \quad (33)$$

$$+\frac{1}{|\mathbf{q}_1-\mathbf{Q}_{\rm COM}+\frac{\mathbf{Q}_{\rm rel}}{2}|}+\frac{1}{|\mathbf{q}_2-\mathbf{Q}_{\rm COM}+\frac{\mathbf{Q}_{\rm rel}}{2}|}|\Psi^{\rm L,S}_{{\rm AB},J}(t)\rangle$$

$$= \frac{2V_{LL,{\rm AB}}+2V_{SS,{\rm AB}}+4S_{\rm LS}(t)V_{\rm LS,AB}}{1+S_{\rm LS}^2}. \quad (34)$$

The explicit analytical form of the nucleus-electron integral $V_{\rm LS,AB}$ in the NEWPMD-QR method was calculated as

$$V_{\rm LS,AB} = -S_{\rm LS}(t)\int d\mathbf{Q}_{\rm COM}\int d\mathbf{Q}_{\rm rel}\int d\mathbf{q}_1$$

$$\frac{\phi_{\rm L}(\mathbf{q}_1,t)\phi_{\rm S}(\mathbf{q}_1,t)\Phi^*_{{\rm AB},J}(\mathbf{Q}_1,\mathbf{Q}_2,t)\Phi_{{\rm AB},J}(\mathbf{Q}_1,\mathbf{Q}_2,t)}{|\mathbf{q}_1-\mathbf{Q}_{\rm COM}-\frac{\mathbf{Q}_{\rm rel}}{2}|} \quad (35)$$

$$= -S_{\rm LS}(t)\int d\mathbf{Q}_{\rm COM}\int d\mathbf{Q}_{\rm rel}\int d\mathbf{q}_1\frac{1}{|\mathbf{q}_1-\mathbf{Q}_{\rm COM}-\frac{\mathbf{Q}_{\rm rel}}{2}|}$$

$$\times\ N_{\rm L}^{-1}N_{\rm S}^{-1}\exp\left[-\frac{(\mathbf{q}-\mathbf{R}_{\rm e}^{\rm L}(t))^2}{4\rho_{\rm L}^2(t)}\right]\exp\left[-\frac{(\mathbf{q}-\mathbf{R}_{\rm e}^{\rm S}(t))^2}{4\rho_{\rm S}^2(t)}\right]$$

$$\times\ N_{{\rm AB},J}^{-2}\exp\left[-\frac{(\mathbf{Q}_{\rm COM}-\mathbf{R}_{\rm COM}(t))^2}{\Omega_{\rm A}^2(t)}-\frac{(Q_{\rm rel}-R_{\rm rel}(t))^2}{4\Omega_{\rm A}^2(t)}\right]Y_{Jm}^2(\chi,\omega) \quad (36)$$

$$= -\frac{S_{\rm LS}(t)}{N_{\rm L}N_{\rm S}N_{{\rm AB},J}^2}\int d\mathbf{Q}_{\rm COM}\int d\mathbf{Q}_{\rm rel}\int d\mathbf{q}_1$$

$$
\begin{aligned}
& \int \frac{\mathrm{d}\mathbf{k}_1}{8\pi^3}\frac{4\pi}{k_1^2}\exp\left[-\mathrm{i}\mathbf{k}_1\cdot(\mathbf{q}_1-\mathbf{Q}_{\mathrm{COM}}-\frac{\mathbf{Q}_{\mathrm{rel}}}{2})\right] \\
& \int \frac{\mathrm{d}\mathbf{k}_2}{\pi^{\frac{3}{2}}}\rho_{\mathrm{L}}^3(t)\exp\left[-\rho_{\mathrm{L}}^2(t)\mathbf{k}_2^2-\mathrm{i}\mathbf{k}_2\cdot(\mathbf{q}_1-\mathbf{R}_{\mathrm{e}}^{\mathrm{L}}(t))\right] \\
& \int \frac{\mathrm{d}\mathbf{k}_3}{\pi^{\frac{3}{2}}}\rho_{\mathrm{S}}^3(t)\exp\left[-\rho_{\mathrm{S}}^2(t)\mathbf{k}_3^2-\mathrm{i}\mathbf{k}_3\cdot(\mathbf{q}_1-\mathbf{R}_{\mathrm{e}}^{\mathrm{S}}(t))\right] \\
& \int \frac{\mathrm{d}\mathbf{k}_4}{\pi^{\frac{3}{2}}}\left(\frac{\Omega_{\mathrm{A}}(t)}{2}\right)^3\exp\left[-\frac{\Omega_{\mathrm{A}}^2(t)\mathbf{k}_4^2}{4}-\mathrm{i}\mathbf{k}_4\cdot(\mathbf{Q}_{\mathrm{COM}}-\mathbf{R}_{\mathrm{COM}}(t))\right] \\
& \exp\left[-\frac{(Q_{\mathrm{rel}}-R_{\mathrm{rel}}(t))^2}{4\Omega_{\mathrm{A}}^2(t)}\right]Y_{Jm}^2(\chi,\omega) \qquad (37)\\
= \; & -\frac{S_{\mathrm{LS}}(t)\rho_{\mathrm{L}}^3(t)\rho_{\mathrm{S}}^3(t)\Omega_{\mathrm{A}}^3(t)}{16\pi^{\frac{13}{2}}N_{\mathrm{L}}N_{\mathrm{S}}N_{\mathrm{AB},J}^2}\int \mathrm{d}\mathbf{Q}_{\mathrm{COM}}\int \mathrm{d}\mathbf{Q}_{\mathrm{rel}}\int \mathrm{d}\mathbf{q}_1 \\
& \int \frac{\mathrm{d}\mathbf{k}_1}{k_1^2}\int \mathrm{d}\mathbf{k}_2\exp\left[-\rho_{\mathrm{L}}^2(t)\mathbf{k}_2^2\right]\int \mathrm{d}\mathbf{k}_3\exp\left[-\rho_{\mathrm{S}}^2(t)\mathbf{k}_3^2\right] \\
& \int \mathrm{d}\mathbf{k}_4\exp\left[-\frac{\Omega_{\mathrm{A}}^2(t)\mathbf{k}_4^2}{4}\right]\exp\left[-\frac{(Q_{\mathrm{rel}}-R_{\mathrm{rel}}(t))^2}{4\Omega_{\mathrm{A}}^2(t)}\right]Y_{Jm}^2(\chi,\omega) \\
& \exp\left[-\mathrm{i}\mathbf{q}_1\cdot(\mathbf{k}_1+\mathbf{k}_2+\mathbf{k}_3)\right]\exp\left[\mathrm{i}\mathbf{Q}_{\mathrm{COM}}\cdot(\mathbf{k}_1-\mathbf{k}_4)\right] \\
& \exp\left[\mathrm{i}\left(\frac{\mathbf{k}_1\cdot\mathbf{Q}_{\mathrm{rel}}}{2}+\mathbf{k}_2\cdot\mathbf{R}_{\mathrm{e}}^{\mathrm{L}}(t)+\mathbf{k}_3\cdot\mathbf{R}_{\mathrm{e}}^{\mathrm{S}}(t)+\mathbf{k}_4\cdot\mathbf{R}_{\mathrm{COM}}(t)\right)\right] \qquad (38)\\
= \; & -\frac{4S_{\mathrm{LS}}(t)\rho_{\mathrm{L}}^3(t)\rho_{\mathrm{S}}^3(t)\Omega_{\mathrm{A}}^3(t)}{\pi^{\frac{1}{2}}N_{\mathrm{L}}N_{\mathrm{S}}N_{\mathrm{AB},J}^2}\int \mathrm{d}\mathbf{Q}_{\mathrm{rel}}\int\frac{\mathrm{d}\mathbf{k}_1}{k_1^2}\int \mathrm{d}\mathbf{k}_2\exp\left[-\rho_{\mathrm{L}}^2(t)\mathbf{k}_2^2\right] \\
& \int \mathrm{d}\mathbf{k}_3\exp\left[-\rho_{\mathrm{S}}^2(t)\mathbf{k}_3^2\right]\int \mathrm{d}\mathbf{k}_4\exp\left[-\frac{\Omega_{\mathrm{A}}^2(t)\mathbf{k}_4^2}{4}\right] \\
& \exp\left[-\frac{(Q_{\mathrm{rel}}-R_{\mathrm{rel}}(t))^2}{4\Omega_{\mathrm{A}}^2(t)}\right]Y_{Jm}^2(\chi,\omega)\delta(\mathbf{k}_1+\mathbf{k}_2+\mathbf{k}_3)\delta(\mathbf{k}_1-\mathbf{k}_4) \\
& \exp\left[\mathrm{i}\left(\mathbf{k}_1\cdot\frac{\mathbf{Q}_{\mathrm{rel}}}{2}+\mathbf{k}_2\cdot\mathbf{R}_{\mathrm{e}}^{\mathrm{L}}(t)+\mathbf{k}_3\cdot\mathbf{R}_{\mathrm{e}}^{\mathrm{S}}(t)+\mathbf{k}_4\cdot\mathbf{R}_{\mathrm{COM}}(t)\right)\right] \qquad (39)\\
= \; & -\frac{4S_{\mathrm{LS}}(t)\rho_{\mathrm{L}}^3(t)\rho_{\mathrm{S}}^3(t)\Omega_{\mathrm{A}}^3(t)}{\pi^{\frac{1}{2}}N_{\mathrm{L}}N_{\mathrm{S}}N_{\mathrm{AB},J}^2}\int \mathrm{d}\mathbf{Q}_{\mathrm{rel}} \\
& \int\frac{\mathrm{d}\mathbf{k}_1}{k_1^2}\int \mathrm{d}\mathbf{k}_2\exp\left[-\rho_{\mathrm{L}}^2(t)\mathbf{k}_2^2\right]\exp\left[-\rho_{\mathrm{S}}^2(t)(\mathbf{k}_1+\mathbf{k}_2)^2\right] \\
& \exp\left[-\frac{\Omega_{\mathrm{A}}^2(t)\mathbf{k}_1^2}{4}\right]\exp\left[-\frac{(Q_{\mathrm{rel}}-R_{\mathrm{rel}}(t))^2}{4\Omega_{\mathrm{A}}^2(t)}\right]Y_{Jm}^2(\chi,\omega) \\
& \exp\left[\mathrm{i}\mathbf{k}_1\cdot\left(\frac{\mathbf{Q}_{\mathrm{rel}}}{2}-\mathbf{R}_{\mathrm{e}}^{\mathrm{S}}(t)+\mathbf{R}_{\mathrm{COM}}(t)\right)+\mathrm{i}\mathbf{k}_2\cdot\left(\mathbf{R}_{\mathrm{e}}^{\mathrm{L}}(t)-\mathbf{R}_{\mathrm{e}}^{\mathrm{S}}(t)\right)\right] \qquad (40)\\
= \; & -\frac{S_{\mathrm{LS}}^2\pi^{\frac{3}{2}}\Omega_{\mathrm{A}}^3(t)}{N_{\mathrm{AB},0}^2}\int \mathrm{d}\mathbf{Q}_{\mathrm{rel}}\exp\left[-\frac{(Q_{\mathrm{rel}}-R_{\mathrm{rel}}(t))^2}{4\Omega_{\mathrm{A}}^2(t)}\right]Y_{Jm}^2(\chi,\omega)
\end{aligned}
$$

$$\times \quad \frac{2\mathrm{erf}\left[\frac{|\mathbf{Q}_{\mathrm{rel}}+\mathbf{R}_{\mathrm{COMLS}}(t)|}{2\sqrt{\Omega_{\mathrm{A}}^2(t)+4\rho_{\mathrm{e}}^2(t)}}\right]}{|\mathbf{Q}_{\mathrm{rel}}+\mathbf{R}_{\mathrm{COMLS}}(t)|}, \tag{41}$$

with

$$\mathbf{R}_{\mathrm{COMLS}}(t) = 2\mathbf{R}_{\mathrm{COM}}(t) - \frac{2(\rho_{\mathrm{L}}^2(t)\mathbf{R}_{\mathrm{e}}^{\mathrm{S}}(t) + \rho_{\mathrm{S}}^2(t)\mathbf{R}_{\mathrm{e}}^{\mathrm{L}}(t))}{\rho_{\mathrm{L}}^2(t) + \rho_{\mathrm{S}}^2(t)} \tag{42}$$

and

$$\rho_{\mathrm{e}}(t) = \frac{\rho_{\mathrm{L}}(t)\rho_{\mathrm{S}}(t)}{\sqrt{\rho_{\mathrm{L}}^2(t) + \rho_{\mathrm{S}}^2(t)}}. \tag{43}$$

The remained integral by $\mathbf{Q}_{\mathrm{rel}}$ in eq.(41) can be performed by expanding the last line as

$$\begin{aligned} & 2\frac{\mathrm{erf}\left[\frac{|\mathbf{Q}_{\mathrm{rel}}+\mathbf{R}_{\mathrm{COMLS}}(t)|}{2\sqrt{\Omega_{\mathrm{A}}^2(t)+4\rho_{\mathrm{e}}^2(t)}}\right]}{|\mathbf{Q}_{\mathrm{rel}}+\mathbf{R}_{\mathrm{COMLS}}(t)|} \\ \simeq \; & \frac{2}{\sqrt{\pi}\sqrt{\Omega_{\mathrm{A}}^2(t)+4\rho_{\mathrm{e}}^2(t)}} - \frac{|\mathbf{Q}_{\mathrm{rel}}+\mathbf{R}_{\mathrm{COMLS}}(t)|^2}{6\sqrt{\pi}\,(\Omega_{\mathrm{A}}^2(t)+4\rho_{\mathrm{e}}^2(t))^{\frac{3}{2}}} + \frac{|\mathbf{Q}_{\mathrm{rel}}+\mathbf{R}_{\mathrm{COMLS}}(t)|^4}{80\sqrt{\pi}\,(\Omega_{\mathrm{A}}^2(t)+4\rho_{\mathrm{e}}^2(t))^{\frac{5}{2}}}. \end{aligned} \tag{44}$$

Then, the nucleus-electron integral finally becomes

$$\begin{aligned} & V_{\mathrm{LS,AB}} = -\frac{S_{\mathrm{LS}}^2\pi^{\frac{3}{2}}\Omega_{\mathrm{A}}^3(t)}{N_{\mathrm{AB},0}^2} \\ \times \; & \left[\frac{8\sqrt{\pi}\Omega_{\mathrm{A}}(t)}{\sqrt{\Omega_{\mathrm{A}}^2(t)+4\rho_{\mathrm{e}}^2(t)}}\left\{2\mathrm{e}^{-\frac{R_{\mathrm{rel}}^2(t)}{4\Omega_{\mathrm{A}}^2(t)}}R_{\mathrm{rel}}(t)\Omega_{\mathrm{A}}(t) + \sqrt{\pi}\left(R_{\mathrm{rel}}^2(t)+2\Omega_{\mathrm{A}}^2(t)\right)\left(1+\mathrm{erf}\left[\frac{R_{\mathrm{rel}}(t)}{2\Omega_{\mathrm{A}}(t)}\right]\right)\right\}\right. \\ - \; & \frac{2\sqrt{\pi}\Omega_{\mathrm{A}}(t)}{3\,(\Omega_{\mathrm{A}}^2(t)+4\rho_{\mathrm{e}}^2(t))^{\frac{3}{2}}}\left\{2\mathrm{e}^{-\frac{R_{\mathrm{rel}}^2(t)}{4\Omega_{\mathrm{A}}^2(t)}}R_{\mathrm{rel}}(t)\Omega_{\mathrm{A}}(t)\left(\mathbf{R}_{\mathrm{COMLS}}^2(t)+R_{\mathrm{rel}}^2(t)+10\Omega_{\mathrm{A}}^2(t)\right)\right. \\ + \; & \sqrt{\pi}\left(R_{\mathrm{rel}}^4(t)+12R_{\mathrm{rel}}^2(t)\Omega_{\mathrm{A}}^2(t)+12\Omega_{\mathrm{A}}^4(t)+\mathbf{R}_{\mathrm{COMLS}}^2(t)\left(R_{\mathrm{rel}}^2(t)+2\Omega_{\mathrm{A}}^2(t)\right)\right) \\ \times \; & \left.\left(1+\mathrm{erf}\left[\frac{R_{\mathrm{rel}}(t)}{2\Omega_{\mathrm{A}}(t)}\right]\right)\right\} \\ + \; & \frac{\sqrt{\pi}\Omega_{\mathrm{A}}(t)}{60\,(\Omega_{\mathrm{A}}^2(t)+4\rho_{\mathrm{e}}^2(t))^{\frac{5}{2}}}\left\{2\mathrm{e}^{-\frac{R_{\mathrm{rel}}^2(t)}{4\Omega_{\mathrm{A}}^2(t)}}R_{\mathrm{rel}}(t)\Omega_{\mathrm{A}}(t)\right. \\ \times \; & \left(3\mathbf{R}_{\mathrm{COMLS}}^4(t)+10\mathbf{R}_{\mathrm{COMLS}}^2(t)\left(R_{\mathrm{rel}}^2(t)+10\Omega_{\mathrm{A}}^2(t)\right)+3\left(R_{\mathrm{rel}}^2(t)+6\Omega_{\mathrm{A}}^2(t)\right)\left(R_{\mathrm{rel}}^2(t)+22\Omega_{\mathrm{A}}^2(t)\right)\right) \end{aligned} \tag{45}$$

$$+ \quad \sqrt{\pi}\left(R_{\mathrm{rel}}^2(t)\left(3\mathbf{R}_{\mathrm{COMLS}}^2(t)+R_{\mathrm{rel}}^2(t)\right)\left(\mathbf{R}_{\mathrm{COMLS}}^2(t)+3R_{\mathrm{rel}}^2(t)\right)\right.$$
$$+ \quad 6\left(\mathbf{R}_{\mathrm{COMLS}}^4(t)+20\mathbf{R}_{\mathrm{COMLS}}^2(t)R_{\mathrm{rel}}^2(t)+15R_{\mathrm{rel}}^4(t)\right)\Omega_{\mathrm{A}}^2(t)$$
$$+ \quad \left.60\left(2\mathbf{R}_{\mathrm{COMLS}}^2(t)+9R_{\mathrm{rel}}^2(t)\right)\Omega_{\mathrm{A}}^4(t)+360\Omega_{\mathrm{A}}(t)^6\right)\left(1+\mathrm{erf}\left[\frac{R_{\mathrm{rel}}(t)}{2\Omega_{\mathrm{A}}(t)}\right]\right)\Bigg\}\Bigg].$$

This expression does not change even if the integrand in eq.(35), $1/|\mathbf{q}_1 - \mathbf{Q}_{\mathrm{COM}} - \mathbf{Q}_{\mathrm{rel}}/2|$, is replaced by $1/|\mathbf{q}_1 - \mathbf{Q}_{\mathrm{COM}} + \mathbf{Q}_{\mathrm{rel}}/2|$ corresponding to the other nucleus.

For the shell-type species in the different nuclear rotational states, we freely optimized $\mathbf{R}_{\mathrm{e}}^{\mathrm{L}}(t)$, $\mathbf{R}_{\mathrm{e}}^{\mathrm{S}}(t)$, $\rho_{\mathrm{L}}(t)$, and $\rho_{\mathrm{S}}(t)$ of the EWPs and $\Omega_{\mathrm{A}}(t)$ of the NWPs to find the minimum of the total molecular energy $E_{\mathrm{tot},J}$ along $R_{\mathrm{rel}}(t)$ with $\mathbf{R}_{\mathrm{COM}}(t) = (0,0,0)$ while all the momenta set to be zero. (Fig.2(a)) We confirmed the following facts from the optimized electronic and nuclear structures of the shell-type species: (i) The position of the EWP centers is determined solely by the COM position of the hydrogen nuclei as $\mathbf{R}_{\mathrm{e}}^{k}(t) = \mathbf{R}_{\mathrm{COM}}(t)$ for both k =L and S. (Supplementary Fig.3) (ii) The width of the L-EWP and S-EWP, $\rho_{\mathrm{L}}(t)$ and $\rho_{\mathrm{S}}(t)$, is always linearly proportional to the H-H bond length $R_{\mathrm{rel}}(t)$: $\rho_{\mathrm{L}}(t) = 0.4061 + 0.347R_{\mathrm{rel}}(t)$ Å and $\rho_{\mathrm{S}}(t) = 0.1955 + 0.210R_{\mathrm{rel}}(t)$ Å. (Supplementary Figs.4 and 11) (iii) The relationship $R_{\mathrm{rel}}(t) \gg \Omega_{\mathrm{A}}(t)$ always holds regardless of the rotational states as far as the stable hydrogen molecule is maintained. (Fig.2(d), Supplementary Fig.1 and Supplementary Table 2) The facts (i) and (ii) indicate that time evolution of the EWPs can be fully specified by dynamics of the NWPs based on the assumptions that the EWP dynamics is much faster than the NWP dynamics and that the EWPs instantly adjust their widths and center positions to the NWP dynamics at each moment, which is the reason why momenta of the EWP center position and width are removed in eq.(3). In other words, unlike the NWPs, the EWPs are not time-evolved variationally in the NEWPMD-QR method. The fact (iii) validates the approximation $\exp[-R_{\mathrm{rel}}^2(t)/4\Omega_{\mathrm{A}}^2(t)] \ll 1$ and thus its neglection in the all energy and normalization factors derived in the NEWPMD-QR method. The above insights and assumptions greatly simplify the derivations of the EOMs through the time-dependent quantum variational principle for the shell-type species, making their real-time

molecular dynamics simulations computationally feasible.

S1C. Time-dependent quantum variational principle for the nuclear-electron wave function with the nuclear quantum rotation

The EOMs to time-evolve $\mathbf{R}_{\mathrm{COM}}(t)$, $R_{\mathrm{rel}}(t)$, $\Omega_{\mathrm{A}}(t)$, $\mathbf{P}_{\mathrm{COM}}(t)$, $P_{\mathrm{rel}}(t)$, and $\Pi_{\mathrm{A}}(t)$ that specify dynamics of both the NWPs and EWPs can be derived through the time-dependent quantum variational principle. The time-dependent quantum variational principle minimizes the action integral for the time-dependent nuclear-electron wave function $\Psi^{\mathrm{L,S}}_{\mathrm{AB},J}(t)$ in eq.(1) defined as

$$\Gamma \equiv \int \mathcal{L} dt = \int dt \langle \Psi^{\mathrm{L,S}}_{\mathrm{AB},J}(t) | i\frac{\partial}{\partial t} - \hat{H} | \Psi^{\mathrm{L,S}}_{\mathrm{AB},J}(t) \rangle, \tag{46}$$

with the quantum Lagrangian $\mathcal{L}$. Since the Hamiltonian operator $\hat{H}$ is composed of the kinetic energy operators of the electrons (eq.(18)) and hydrogen nuclei (eq.(26)) as well as the three electrostatic interaction energy operators of electron-electron (eq.(21)), nucleus-nucleus (eq.(29)) and nucleus-electron (eq.(32)), $\langle \Psi^{\mathrm{L,S}}_{\mathrm{AB},J}(t) | \hat{H} | \Psi^{\mathrm{L,S}}_{\mathrm{AB},J}(t) \rangle = E_{\mathrm{tot},J}$ from the definition, eq.(17). The time-dependent variational principle, $\delta\Gamma/\delta\mathbf{R}_{\mathrm{COM}}(t) = 0$, etc., yields the EOMs,

$$\sum_k -2\mathrm{Im} \left\langle \frac{\partial \Psi^{\mathrm{L,S}}_{\mathrm{AB},J}(t)}{\partial X_I(t)} | \frac{\partial \Psi^{\mathrm{L,S}}_{\mathrm{AB},J}(t)}{\partial X_k(t)} \right\rangle \dot{X}_k(t) = \frac{\partial E_{\mathrm{tot},J}}{\partial X_I(t)}, \tag{47}$$

where $\mathbf{X}(t) = \{\mathbf{R}_{\mathrm{COM}}(t), R_{\mathrm{rel}}(t), \Omega_{\mathrm{A}}(t), \mathbf{P}_{\mathrm{COM}}(t), P_{\mathrm{rel}}(t), \Pi_{\mathrm{A}}(t)\}$. The explicit forms of these EOMs are written as

$$-2\dot{\mathbf{P}}_{\mathrm{COM}}(t) = \frac{\partial E_{\mathrm{tot},J}}{\partial \mathbf{R}_{\mathrm{COM}}(t)}, \tag{48}$$

$$
\begin{aligned}
- & \frac{R_{\rm rel}^4(t)+12\Omega_{\rm A}^4(t)}{2(R_{\rm rel}^2(t)+2\Omega_{\rm A}^2(t))^2}\dot{P}_{\rm rel}(t)-\frac{2R_{\rm rel}^3(t)\Pi_{\rm A}(t)}{(R_{\rm rel}^2(t)+2\Omega_{\rm A}^2(t))^2}\dot{\Omega}_{\rm A}(t) \\
& -\frac{2R_{\rm rel}^3(t)\Omega_{\rm A}(t)}{(R_{\rm rel}^2(t)+2\Omega_{\rm A}^2(t))^2}\dot{\Pi}_{\rm A}(t)=\frac{\partial E_{{\rm tot},J}}{\partial R_{\rm rel}(t)},
\end{aligned} \tag{49}
$$

$$
\begin{aligned}
& \frac{2R_{\rm rel}^3(t)\Pi_{\rm A}(t)}{(R_{\rm rel}^2(t)+2\Omega_{\rm A}^2(t))^2}\dot{R}_{\rm rel}(t)+\frac{4R_{\rm rel}^3(t)\Omega_{\rm A}(t)}{(R_{\rm rel}^2(t)+2\Omega_{\rm A}^2(t))^2}\dot{P}_{\rm rel}(t) \\
& -\left\{6-\frac{2R_{\rm rel}^4(t)}{(R_{\rm rel}^2(t)+2\Omega_{\rm A}^2(t))^2}\right\}\dot{\Pi}_{\rm A}(t)=\frac{\partial E_{{\rm tot},J}}{\partial \Omega_{\rm A}(t)},
\end{aligned} \tag{50}
$$

$$
2\dot{\mathbf{R}}_{\rm COM}(t)=\frac{\partial E_{{\rm tot},J}}{\partial \mathbf{P}_{\rm COM}(t)}, \tag{51}
$$

$$
\frac{R_{\rm rel}^4(t)+12\Omega_{\rm A}^4(t)}{2(R_{\rm rel}^2(t)+2\Omega_{\rm A}^2(t))^2}\dot{R}_{\rm rel}(t)+\frac{4R_{\rm rel}^3(t)\Omega_{\rm A}(t)}{(R_{\rm rel}^2(t)+2\Omega_{\rm A}^2(t))^2}\dot{\Omega}_{\rm A}(t)=\frac{\partial E_{{\rm tot},J}}{\partial P_{\rm rel}(t)}, \tag{52}
$$

and

$$
\frac{2R_{\rm rel}^3(t)\Omega_{\rm A}(t)}{(R_{\rm rel}^2(t)+2\Omega_{\rm A}^2(t))^2}\dot{R}_{\rm rel}(t)+\left\{6-\frac{2R_{\rm rel}^4(t)}{(R_{\rm rel}^2(t)+2\Omega_{\rm A}^2(t))^2}\right\}\dot{\Omega}_{\rm A}(t)=\frac{\partial E_{{\rm tot},J}}{\partial \Pi_{\rm A}(t)}. \tag{53}
$$

It should be mentioned that, since the difference in $\Psi_{{\rm AB},J}^{\rm L,S}(t)$ among the shell-type species is only the spherical harmonics $Y_{Jm}(\chi,\omega)$, the terms on the left-hand side of eqs.(48)-(53) are the same regardless of the shell-type species (J-independent), and that it is the total molecular energy $E_{{\rm tot},J}$ that distinguishes the shell-type species. The EOMs (48)-(53) can be drastically simplified if we adopt the reasonable approximations $R_{\rm rel}(t) \gg \Omega_{\rm A}(t)$ and $P_{\rm rel}(t) \gg \Pi_{\rm A}(t)$, which actually always holds during all molecular dynamics simulations reported in this Article, resulting in

$$
\dot{\mathbf{P}}_{\rm COM}(t)=-\frac{1}{2}\frac{\partial E_{{\rm tot},J}}{\partial \mathbf{R}_{\rm COM}(t)}, \tag{54}
$$

$$\dot{P}_{\rm rel}(t) = -2\frac{\partial E_{{\rm tot},J}}{\partial R_{\rm rel}(t)}, \tag{55}$$

$$\dot{\Pi}_{\rm A}(t) = -\frac{1}{4}\frac{\partial E_{{\rm tot},J}}{\partial \Omega_{\rm A}(t)}, \tag{56}$$

$$\dot{\mathbf{R}}_{\rm COM}(t) = \frac{1}{2}\frac{\partial E_{{\rm tot},J}}{\partial \mathbf{P}_{\rm COM}(t)} = \frac{\mathbf{P}_{\rm COM}(t)}{M}, \tag{57}$$

$$\dot{R}_{\rm rel}(t) = 2\frac{\partial E_{{\rm tot},J}}{\partial P_{\rm rel}(t)} = \frac{P_{\rm rel}(t)}{M}, \tag{58}$$

and

$$\dot{\Omega}_{\rm A}(t) = \frac{1}{4}\frac{\partial E_{{\rm tot},J}}{\partial \Pi_{\rm A}(t)} = \frac{\Pi_{\rm A}(t)}{M}. \tag{59}$$

It is remarkable that the EOMs (54)-(59) are universal and can be systematically applicable not only to the current four shell-type species (para, ortho, para-2, and ortho-2) but also to another shell-type species possessing higher-energy nuclear quantum rotation. The NEWPMD-QR method is the first computational method to calculate real-time molecular dynamics on a nuclear excited state not on an electronically excited state.

S1D. Interaction potential energy between a rotationally-excited hydrogen molecule and carbon nano tube

In order to simulate real-time collision dynamics of the shell-type species with a single-walled carbon nano tube (SWCNT), CNT(15,0) of the diameter $D_{\rm CNT}$ = 11.71 Å, we calculated the interaction potential energy between the shell-type species and CNT(15,0), $E_{{\rm CNT},J}(r, \theta)$ in which θ denotes the angle between the H-H molecular axis and the x-axis.

First, the total energy of a classical hydrogen molecule possessing two point nuclei and CNT(15,0), $E_{\mathrm{CNT}}(r, \chi_{\mathrm{CNT}})$, was calculated by a density functional theory (DFT) as a two dimensional (2D) function of the distance r between the COM of the hydrogen molecule and the CNT(15,0) surface and the angle χ_{CNT} from the x-axis.(Supplementary Fig.8) The center of the six-carbon ring of CNT(15,0) was chosen as a main adsorption site. The DFT computations were done with the PBE density functional and PAW pseudopotentials using the VASP package.[1] The Grimme D2 correction (PBE-D2) was employed and the scaling parameter s_6 in the PBE-D2 scheme was set as 0.6 with the other parameters remained as default values.[2] The energy cutoff is 400 eV, and the unit cell length of the periodic CNT(15,0) was set as 8.52 Å along the direction parallel to the CNT(15,0) axis (z-axis). The vacuum of approximately 2 nm and 1 nm was inserted to the x- and y-directions, respectively. The analytical total energy function,

$$E_{\mathrm{CNT}}(r, \chi_{\mathrm{CNT}}) = a \left[\left(\frac{b(\chi_{\mathrm{CNT}})}{r} \right)^{6.8} - \left(\frac{b(\chi_{\mathrm{CNT}})}{r} \right)^{5.5} \right], \tag{60}$$

was obtained by fitting the DFT-calculated total energy data points with the two coefficients a and $b(\chi_{\mathrm{CNT}})$ being adjusted. While the coefficient $a = 8250.86$ K was found to be almost independent of χ_{CNT}, the coefficient $b(\chi_{\mathrm{CNT}})$ becomes a function of χ_{CNT} (Supplementary Fig.9):

$$b(\chi_{\mathrm{CNT}}) = 2.559 - \frac{\cos^2 \chi_{\mathrm{CNT}}}{10.80}. \tag{61}$$

The interaction potential energy between the shell-type species and CNT(15,0), $E_{\mathrm{CNT},J}(r, \theta)$, was calculated by further averaging the total energy function $E_{\mathrm{CNT}}(r, \chi_{\mathrm{CNT}})$ with respect to the rotational degrees of freedom,

$$E_{\mathrm{CNT},J}(r, \theta) = \frac{1}{N^2_{\mathrm{CNT},J}} \int_0^{\pi} \sin \chi_{\mathrm{CNT}} d\chi_{\mathrm{CNT}} \int_0^{2\pi} d\omega_{\mathrm{CNT}} E_{\mathrm{CNT}}(r, \chi_{\mathrm{CNT}}) Y^2_{Jm}(\chi_{\mathrm{CNT}} - \theta, \omega_{\mathrm{CNT}}). \tag{62}$$

Here, the J-dependent normalization factors are

$$N_{\mathrm{CNT},0} = 2\sqrt{\pi}, \tag{63}$$

$$N_{\mathrm{CNT},1} = \sqrt{2\pi(1-\cos 2\theta)/3}, \tag{64}$$

$$N_{\mathrm{CNT},2} = 2\sqrt{10\pi/(55-20\cos 2\theta - 3\cos 4\theta)}, \tag{65}$$

and

$$N_{\mathrm{CNT},3} = 4\sqrt{14\pi/(238-91\cos 2\theta - 14\cos 4\theta - 5\cos 6\theta)}. \tag{66}$$

$Y^2_{Jm}(\chi_{\mathrm{CNT}} - \theta, \omega_{\mathrm{CNT}})$ corresponds to the probability weight to sample the angular wave function intrinsic to each shell-type species; it is the only difference among the shell-type species that distinguishes para, ortho, para-2, and ortho-2. For instance, para has $Y^2_{00}(\chi_{\mathrm{CNT}} - \theta, \omega_{\mathrm{CNT}}) = 1$, requiring the completely spherical angular sampling. The explicit forms of the interaction potential energy $E_{\mathrm{CNT},J}(r,\theta)$ finally become

$$E_{\mathrm{CNT},0}(r,\theta) = a\left(\frac{549.60628}{r^{6.8}} - \frac{164.45135}{r^{5.5}}\right), \tag{67}$$

$$\begin{aligned} E_{\mathrm{CNT},1}(r,\theta) &= \frac{a}{r^{12.3}(-3+\cos 2\theta)} \\ &\times \left\{r^{6.8}(493.35405 - 181.82782\cos 2\theta)\right. \\ &\quad \left. + r^{5.5}(-1648.8188 + 621.05063\cos 2\theta)\right\}, \end{aligned} \tag{68}$$

$$E_{\mathrm{CNT},2}(r,\theta) = \frac{5a}{r^{12.3}(-55+20\cos 2\theta + 3\cos 4\theta)} \tag{69}$$

$$\times \left\{ r^{6.8}(1808.9648 - 727.31129\cos 2\theta - 67.760633\cos 4\theta) \right.$$
$$\left. + r^{5.5}(-6045.6691 + 2484.2025\cos 2\theta + 201.35079\cos 4\theta) \right\},$$

and

$$E_{\mathrm{CNT},3}(r,\theta) = \frac{112a}{r^{12.3}(-238 + 91\cos 2\theta + 14\cos 4\theta + 5\cos 6\theta)} \tag{70}$$
$$\times \left\{ r^{5.5}(-1167.9133 + 504.60363\cos 2\theta + 41.948080\cos 4\theta + 19.797805\cos 6\theta) \right.$$
$$\left. + r^{6.8}(349.45911 - 147.73510\cos 2\theta - 14.116799\cos 4\theta - 6.1570528\cos 6\theta) \right\}.$$

The obtained interaction potential energy $E_{\mathrm{CNT},J}(r,\theta)$ is also graphically shown in Supplementary Figs.5 and 10. While para exhibits the uniform interaction potential energy along θ (Supplementary Fig.5(a,e)), the interaction potential energy of the other shell-type species shows the clear θ-dependence according to the rotationally excited states.(Supplementary Fig.5(f-h)) The orientational preference demonstrated by the θ-dependence (Supplementary Fig.10) indicates that the degeneracy of the rotationally excited states is lifted due to the anisotropic interaction with the CNT(15,0) surface; $(2J+1)$ of the m-sublevels would be equally populated for each J in the spherical harmonics $Y_{Jm}(\chi,\omega)$ of eq.(14) without any external interaction. Breaking of the degeneracy lets each shell-type species have the preferred orientation upon the adsorption, inducing the hindered rotation around the potential energy minimum. Actually, all of ortho, para-2, and ortho-2 tend to form edge-on attaching to the CNT(15,0) surface. (See the blue regions in Supplementary Fig.10(d-f)) In the case of the G-NEWPMD method, sampling over the angular degrees of freedom performed in eq.(62) is not required, and the total energy function $E_{\mathrm{CNT}}(r,\theta)$ in eq.(60) with χ_{CNT} replaced by θ directly becomes as the interaction potential energy.

S1E. Equations of motion

The anisotropic interaction with the CNT(15,0) surface breaks the spatial translational symmetry on the cross section of CNT(15,0), enabling the introduction of a laboratory coordinate system. Therefore, by transforming the EOMs (54)-(59), we derived the final EOMs as

$$\dot{\mathbf{P}}_{\mathrm{COM}}(t) = -\frac{1}{2}\frac{\partial E_{\mathrm{tot},J}}{\partial \mathbf{R}_{\mathrm{COM}}(t)} - \frac{1}{2}\frac{\partial E_{\mathrm{CNT},J}(\frac{D_{\mathrm{CNT}}}{2} - r, \theta)}{\partial \mathbf{R}_{\mathrm{COM}}(t)}, \tag{71}$$

$$\dot{\mathbf{P}}_{\mathrm{rel}}(t) = -2\frac{\partial E_{\mathrm{tot},J}}{\partial R_{\mathrm{rel}}(t)}\frac{\mathbf{R}_{\mathrm{rel}}(t)}{R_{\mathrm{rel}}(t)} - 2\frac{\partial E_{\mathrm{CNT},J}(\frac{D_{\mathrm{CNT}}}{2} - r, \theta)}{\partial \mathbf{R}_{\mathrm{rel}}(t)}, \tag{72}$$

$$\dot{\Pi}_{\mathrm{A}}(t) = -\frac{1}{4}\frac{\partial E_{\mathrm{tot},J}}{\partial \Omega_{\mathrm{A}}(t)}, \tag{73}$$

$$\dot{\mathbf{R}}_{\mathrm{COM}}(t) = \frac{\mathbf{P}_{\mathrm{COM}}(t)}{M}, \tag{74}$$

$$\dot{\mathbf{R}}_{\mathrm{rel}}(t) = \frac{\mathbf{P}_{\mathrm{rel}}(t)}{M}, \tag{75}$$

and

$$\dot{\Omega}_{\mathrm{A}}(t) = \frac{\Pi_{\mathrm{A}}(t)}{M}. \tag{76}$$

Note that we set the origin as the center of CNT(15,0) in the above EOMs by replacing r with $D_{\mathrm{CNT}}/2 - r$ as seen in $E_{\mathrm{CNT},J}(D_{\mathrm{CNT}}/2 - r, \theta)$.

Species	Total energy (K)	Energy difference (K)	Experiment (K)	Population ratio
para	-311028.299	0	0	1.00
ortho	-310856.464	171.8	170.5 (0.76 %)	3.81
para-2	-310514.103	514.2	509.9 (0.84 %)	0.382
ortho-2	-310003.798	1024.5	1015.2 (0.92 %)	0.125

Supplementary Table 1. Molecular energy and population. Total molecular energies of para, ortho, para-2, and ortho-2 with their energy differences and their population ratios at 200 K. The numbers in the parentheses denote the deviation from the experimental values.[3,4]

Species	H-H length(Å)	quantum H-H length(Å)	NWP width (Å)
Gaussian	0.74551	0.76575	0.06488
para	0.74349	0.76207	0.05914
ortho	0.74445	0.76303	0.05917
para-2	0.74635	0.76492	0.05924
ortho-2	0.74921	0.76777	0.05933

Supplementary Table 2. Molecular structures: Nuclei. H-H bond length and NWP width of Gaussian and the shell-type species (para, ortho, para-2, and ortho-2). In comparison with the normal H-H bond length R_{rel}, the quantum H-H length with the NWP width taken into consideration is also listed.

Species	L-EWP width (Å)	S-EWP width (Å)
Gaussian	0.66683	0.35777
para	0.66418	0.35167
ortho	0.66451	0.35187
para-2	0.66518	0.35227
ortho-2	0.66617	0.35288

Supplementary Table 3. Molecular structures: Electrons. L-EWP width and S-EWP width of Gaussian and the shell-type species (para, ortho, para-2, and ortho-2).

Species	CNT-0 (cm^{-1})	CNT-45 (cm^{-1})	CNT-90 (cm^{-1})	Experiment (cm^{-1})
Gaussian	4619.488	4617.452	4619.488	N/A (11.0 %)
para	4232.410	4232.410	4232.410	4161.13 cm^{-1} (1.7 %)
ortho	4227.574	4227.320	4227.574	4155.20 cm^{-1} (1.7 %)
para-2	4218.158	4217.649	4218.158	4143.39 cm^{-1} (1.8 %)
ortho-2	4203.652	4203.398	4203.652	4125.83 cm^{-1} (1.9 %)

Supplementary Table 4. H-H vibrational frequencies. H-H vibrational frequencies of Gaussian and the shell-type species (para, ortho, para-2, and ortho-2) obtained from the peak frequencies of their H-H vibrational power spectra displayed in Fig.5(a-d). The numbers in the parentheses denote the deviation between the CNT-0 and experimental values.[3,5]

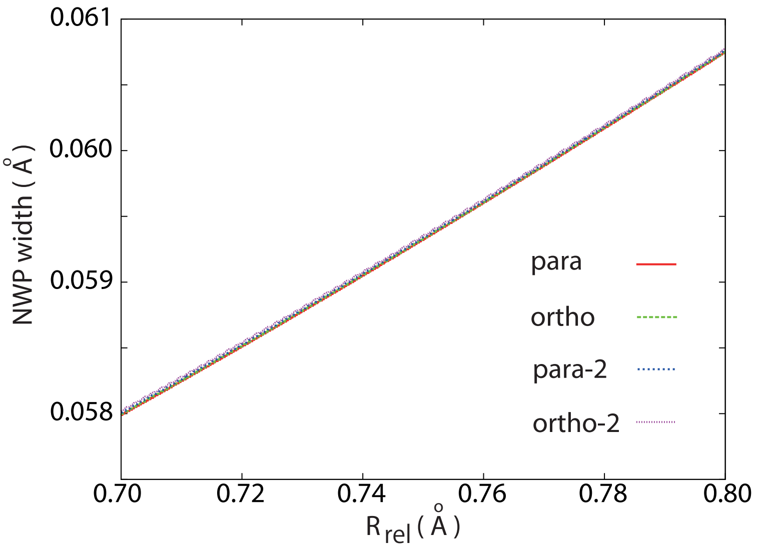

Supplementary Fig.1. NWP width of para, ortho, para-2, and ortho-2. NWP width of the shell-type species as a function of R_{rel}.

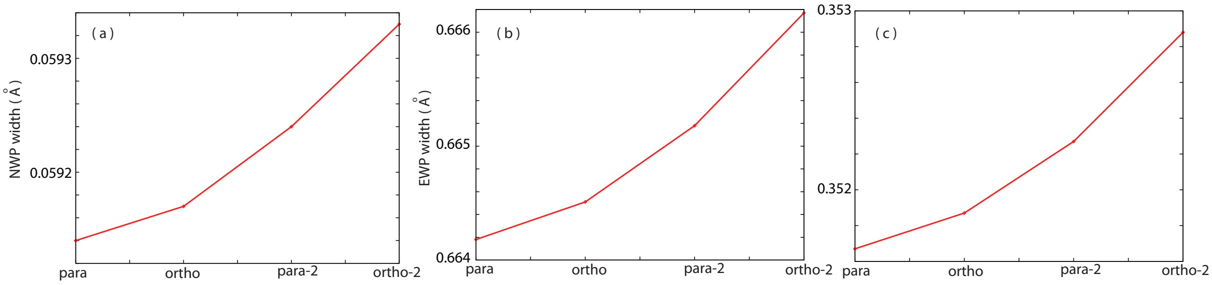

Supplementary Fig.2. Stable molecular structures. (a) NWP width Ω_{A} at the energy minima shown in Fig.2(a). (b) L-EWP and (c) S-EWP width, ρ_{L} and ρ_{S}, at the energy minima shown in Fig.2(a), respectively.

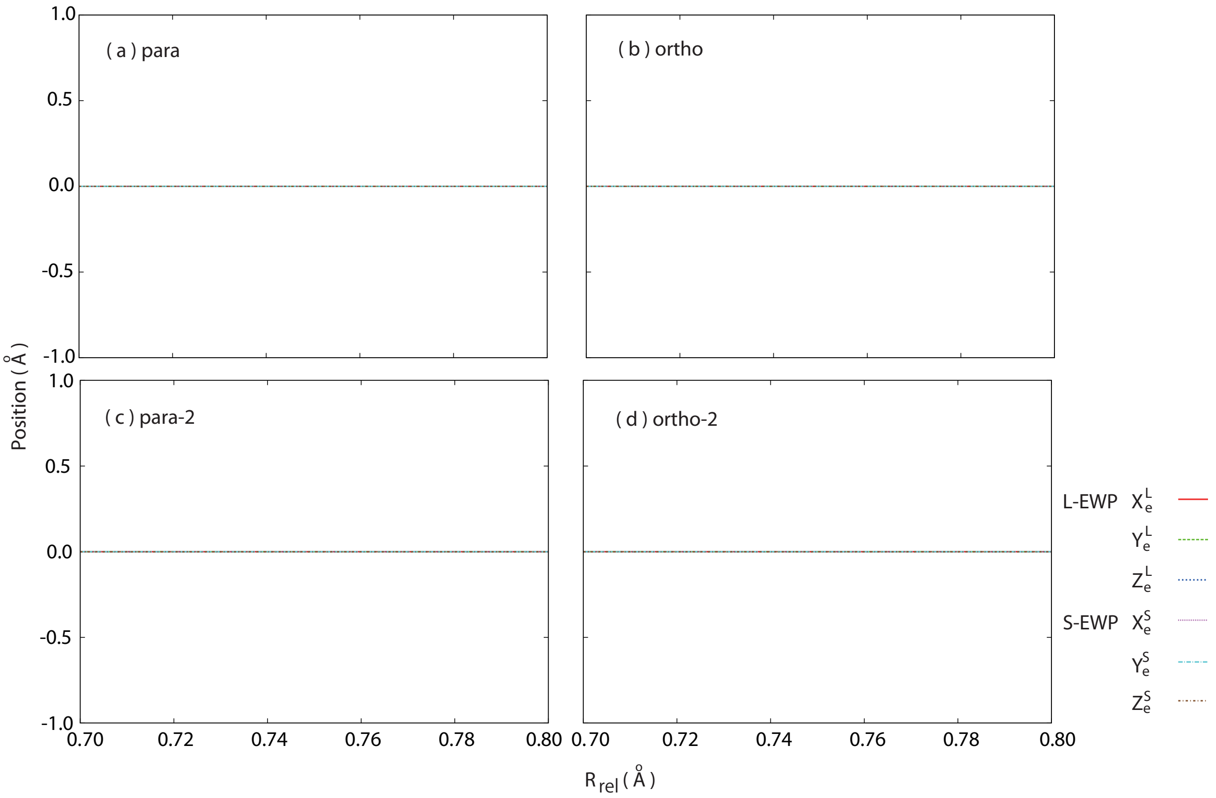

Supplementary Fig.3. Center positions of L-EWPs and S-EWPs of para, ortho, para-2, and ortho-2. Optimized center position of L-EWPs and S-EWPs, $\mathbf{R}_{\rm e}^{\rm L} = (X_{\rm e}^{\rm L}, Y_{\rm e}^{\rm L}, Z_{\rm e}^{\rm L})$ and $\mathbf{R}_{\rm e}^{\rm S} = (X_{\rm e}^{\rm S}, Y_{\rm e}^{\rm S}, Z_{\rm e}^{\rm S})$, of para (a), ortho (b), para-2 (c), and ortho-2 (d) as a function of $R_{\rm rel}$.

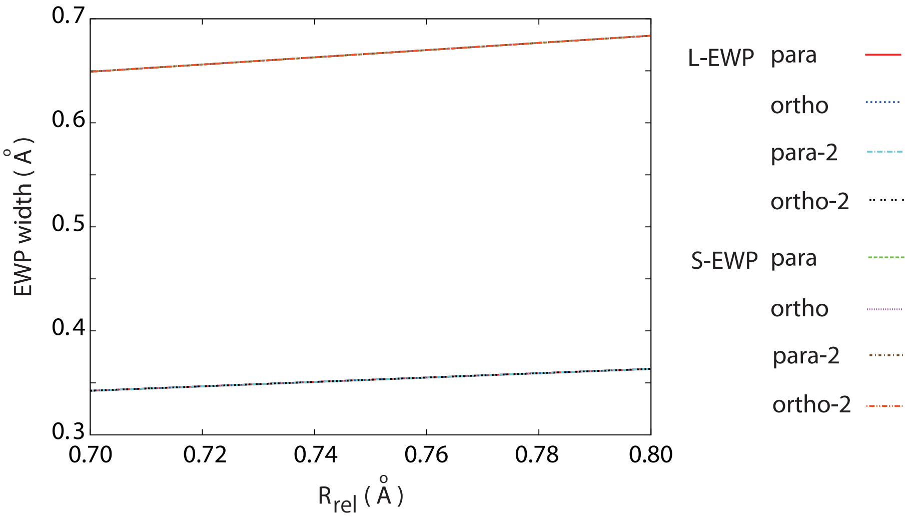

Supplementary Fig.4. EWP width of para, ortho, para-2, and ortho-2. L-EWP and S-EWP width, $\rho_{\rm L}$ and $\rho_{\rm S}$, of the shell-type species as a function of $R_{\rm rel}$. The dependence of the EWP width on $R_{\rm rel}$ is identical regardless of the shell-type species.

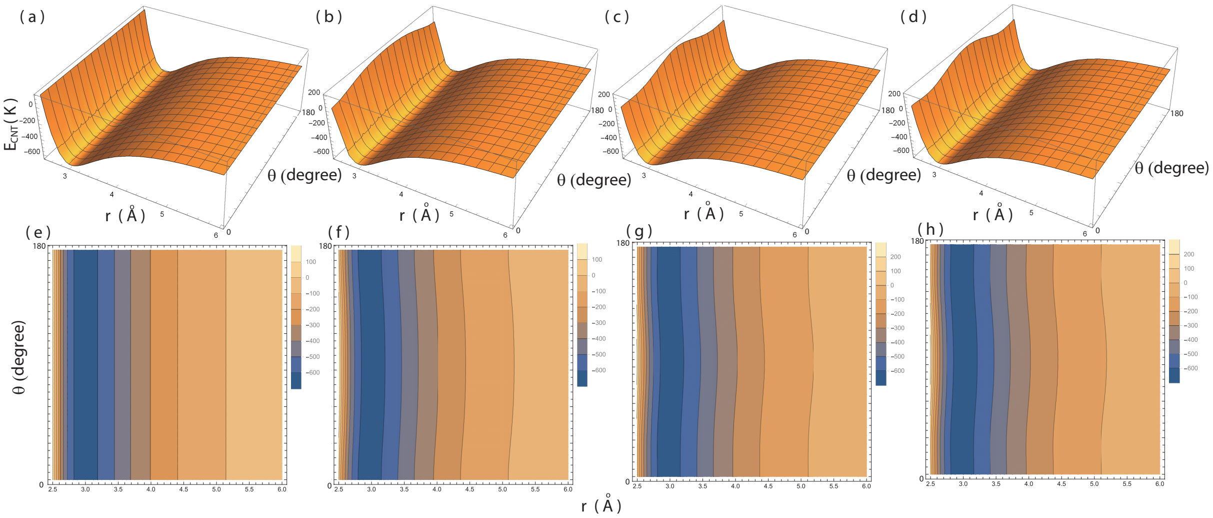

Supplementary Fig.5. Interaction potential energy $\mathbf{E_{CNT,J}(r, \theta)}$. The three-dimensional interaction potential energy $E_{\mathrm{CNT},J}(r,\theta)$ is displayed as a function of the distance r between the COM of the hydrogen molecule and the CNT(15,0) surface and the angle θ of the molecular axis from the x-axis for para ($J = 0$) (a), ortho ($J = 1$) (b), para-2 ($J = 2$) (c), and ortho-2 ($J = 3$) (d). Their 2D contour plots are also drawn for para (e), ortho (f), para-2 (g), and ortho-2 (h).

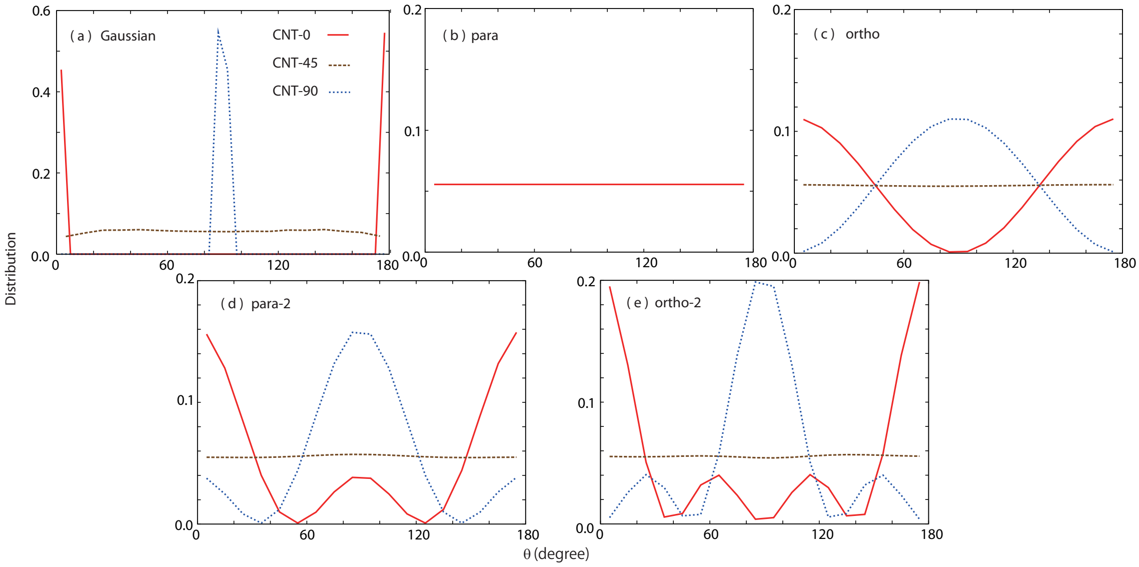

Supplementary Fig.6. Orientational distributions of hydrogen molecules during the collision dynamics. Distributions of the time-dependent angle $\theta(t)$ between the molecular axis and the CNT(15,0) surface for Gaussian (a), para (b), ortho (c), para-2 (d) and ortho-2 (e) taking account of the species-dependent nuclear delocalization. The initial collision angles were set as 0 degree (red line) (CNT-0), 45 degree (brown line) (CNT-45), and 90 degree (blue line) (CNT-90). Since the molecular axis itself does not change during the dynamics in CNT-0 and CNT-90, their orientational distributions simply reflect the nuclear distribution at the fixed molecular axis along the x- and y-axes, respectively. The orientational distributions of para are always uniform due to the completely spherical nuclear wave function. The active orientational dynamics taking place in CNT-45 made the orientational distributions of CNT-45 almost uniform in any species. Such similarity in the orientational distributions could be the origin of the similarity observed in many physical and chemical properties of para-hydrogen and ortho-hydrogen.

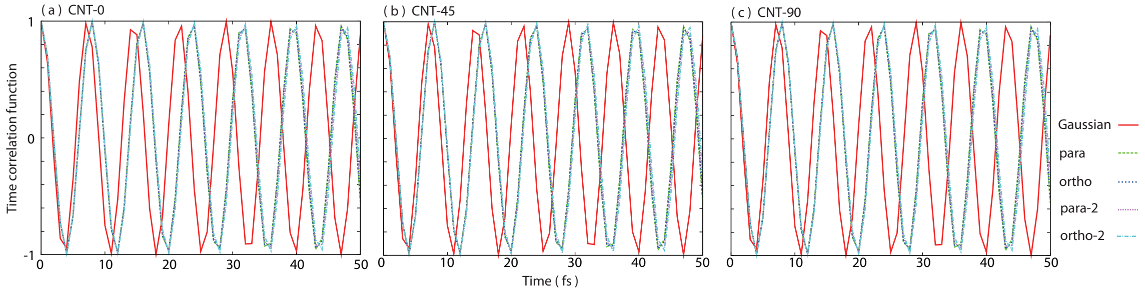

Supplementary Fig.7. TCFs of the time-dependent H-H bond length. TCFs of $R_{\mathrm{rel}}(t)$ during the collision dynamics to CNT(15,0) started with the collision angles between the molecular axis and the CNT surface set as 0 degree (a) (CNT-0), 45 degree (b) (CNT-45), and 90 degree (c) (CNT-90). The TCFs of Gaussian oscillates faster than the TCFs of the shell-type species reflecting its higher H-H vibrational frequency. (Fig.5(e)) Although the all TCFs of the shell-type species seem identical in the shown initial time region, their H-H vibrational frequencies are actually more red-shifted with the higher rotational excitation. (Fig.5(e))

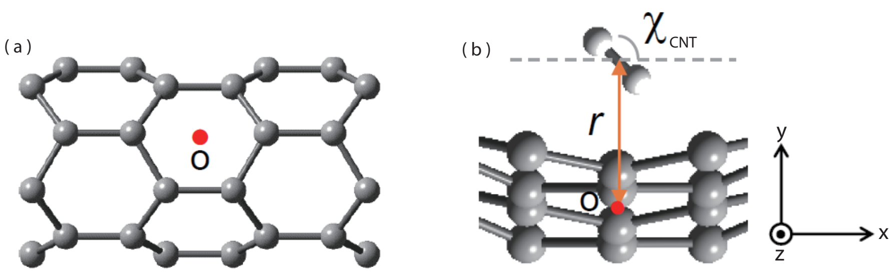

Supplementary Fig.8. Geometrical definition of r and χ_{CNT} in the total energy function $\mathbf{E}_{\mathrm{CNT}}(\mathbf{r}, \chi_{\mathrm{CNT}})$. (a) Adsorption site of a hydrogen molecule on the CNT(15,0) surface. It was set as the center of the six-carbon ring denoted as O (red point). (b) Definitions of the distance r between the COM of the hydrogen molecule and the adsorption site on the CNT(15,0) surface as well as the angle χ_{CNT} from the x-axis.

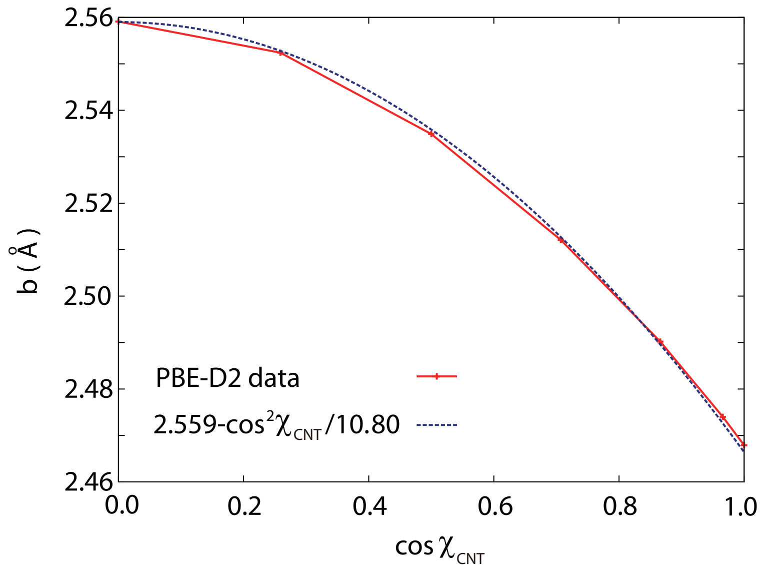

Supplementary Fig.9 Angle-dependent coefficient b($\chi_{\rm CNT}$) in the total energy function $\mathbf{E}_{\rm CNT}(\mathbf{r}, \chi_{\rm CNT})$. The coefficient $b(\chi_{\rm CNT})$ depends on $\chi_{\rm CNT}$. All the numerical data of the total energy computed by the PBE-D2 method were well fitted with $b(\chi_{\rm CNT}) = 2.559 - \cos^2 \chi_{\rm CNT}/10.80$.

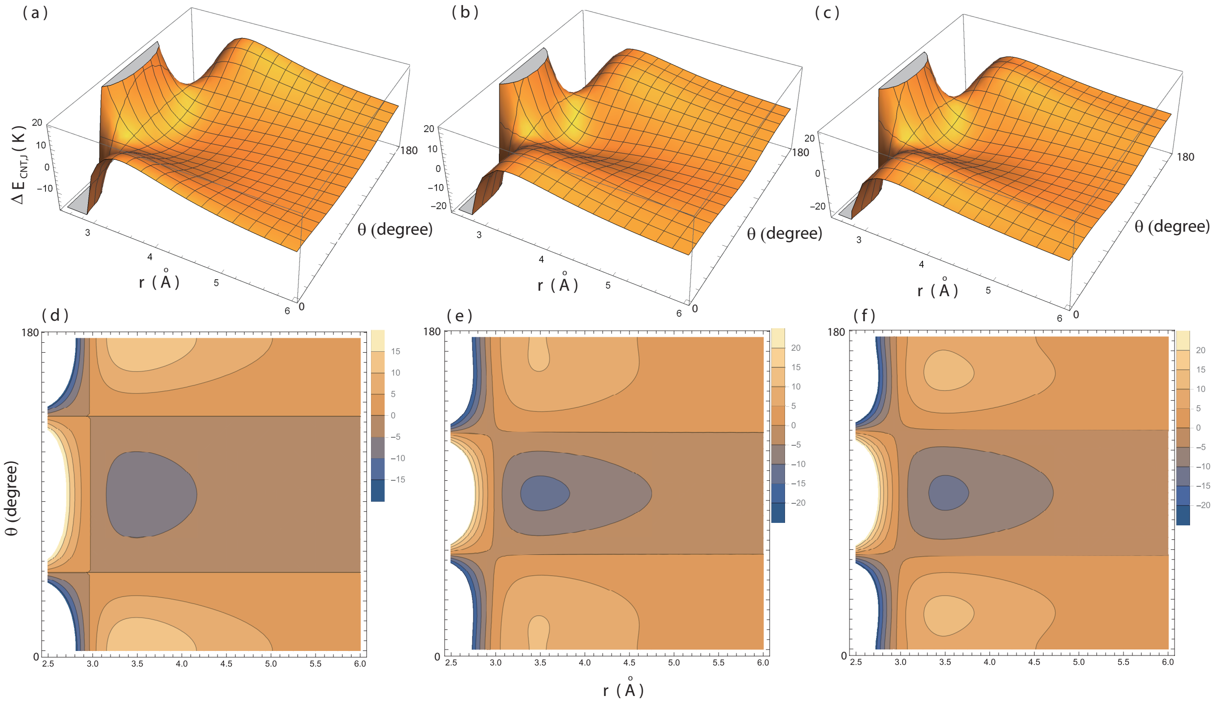

Supplementary Fig.10. Difference between the interaction potential energy of para and the other three shell-type species (ortho, para-2, and ortho-2). The difference in the three-dimensional interaction potential energy, $\Delta E_{\mathrm{CNT},J} = E_{\mathrm{CNT},J}(r,\theta) - E_{\mathrm{CNT},0}(r,\theta)$, is displayed as a function of the distance r between the COM of the hydrogen molecule and the CNT(15,0) surface and the angle θ of the molecular axis from the x-axis for $\Delta E_{\mathrm{CNT},1}$ (a), $\Delta E_{\mathrm{CNT},2}$ (b), and $\Delta E_{\mathrm{CNT},3}$ (c). Their 2D contour plots are also drawn as a 2D contour plot for $\Delta E_{\mathrm{CNT},1}$ (d), $\Delta E_{\mathrm{CNT},2}$ (e), and $\Delta E_{\mathrm{CNT},3}$ (f).

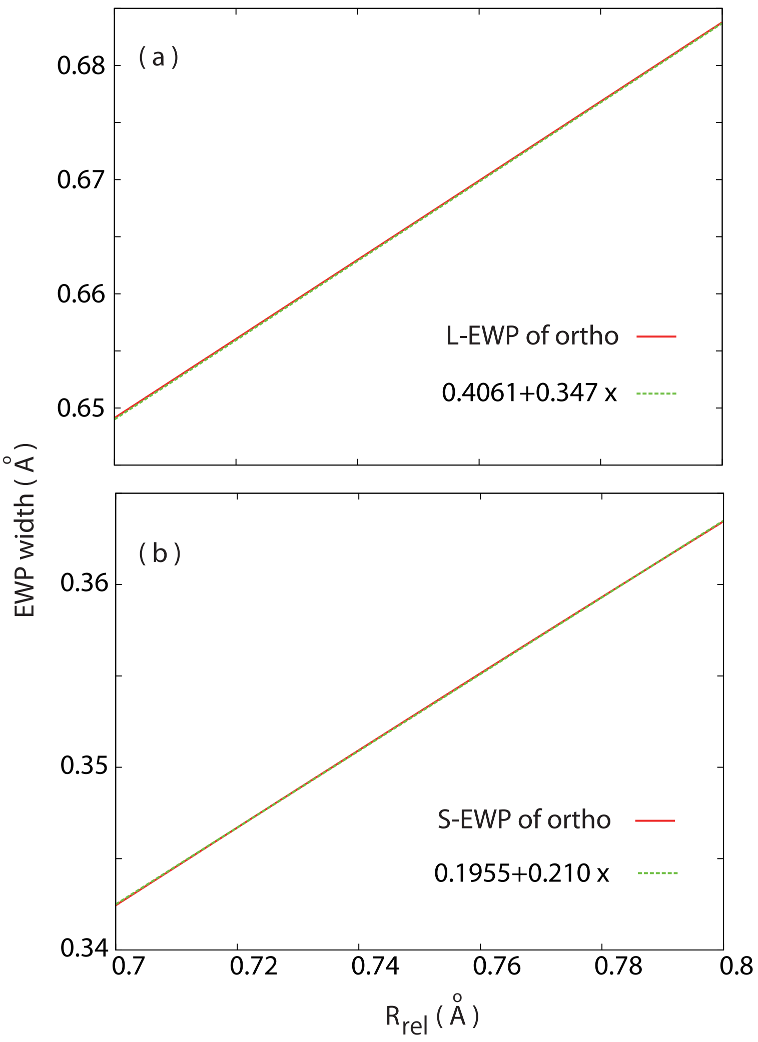

Supplementary Fig.11. EWP width fitted by a linear function of $\mathbf{R}_{\text{rel}}$. (a) L-EWP width of ortho fitted by the linear function $0.4061 + 0.347R_{\text{rel}}$. (b) S-EWP width of ortho fitted by the linear function $0.1955 + 0.210R_{\text{rel}}$. Since all the shell-type species have the same dependence of the EWP width on R_{rel} as shown in Supplementary Fig.4, we picked up only ortho here.

Supplementary References

(1) Kresse, G.; Furthmüller, J. Efficient Iterative Schemes for Ab Initio Total-Energy Calculations using a Plane-Wave Basis Set. *Phys. Rev. B* **1996**, *54*, 11169–11186.

(2) Faginas-Lago, N.; Yeni, D.; Huarte, F.; Wang, Y.; Alcami, M.; Martin, F. Adsorption of Hydrogen Molecules on Carbon Nanotubes using Quantum Chemistry and Molecular Dynamics. *J. Phys. Chem. A,* **2016**, *120*, 6451–6458.

(3) Wolniewicz, L. Vibrational-Rotational Study of the Electronic Ground State of the Hydrogen Molecule. *J. Chem. Phys.* **1966**, *45*, 515–523.

(4) Silvera, I. F. The Solid Molecular Hydrogens in the Condensed Phase: Fundamentals and Static Properties. *Rev. Mod. Phys.* **1980**, *52*, 393.

(5) Karl, G.; Poll, J. D. On the Quadrupole Moment of the Hydrogen Molecule. *J. Chem. Phys.* **1967**, *46*, 2944–2950.
