# Supplementary material for: Real-time hydrogen molecular dynamics satisfying the nuclear spin statistics of a quantum rotor
Source: Commun Chem. 2022 Dec 3;5:168. doi: 10.1038/s42004-022-00788-z (PMC9814564; doi:10.1038/s42004-022-00788-z)
Supplement: Supplementary file 3 — Description of Additional Supplementary Files [file 42004_2022_788_MOESM3_ESM.pdf]

Description of Additional Supplementary Files

File name: Supplementary Movie 1

Description: Real-time collision dynamics of Gaussian showing the delocalized nuclei and nodes. The initial collision angle between the molecular axis and the CNT(15,0) surface was set as 45 degree. See Fig.3 (C,D,I,J).

File name: Supplementary Movie 2

Description: Real-time collision dynamics of para showing the species-dependent delocalized nuclei. See Fig.3 (C,D,I,J).

File name: Supplementary Movie 3

Description: Real-time collision dynamics of ortho showing the species-dependent delocalized nuclei and nodes. The initial collision angle between the molecular axis and the CNT(15,0) surface was set as 45 degree. See Fig.3 (C,D,I,J).

File name: Supplementary Movie 4

Description: Real-time collision dynamics of para-2 showing the species-dependent delocalized nuclei and nodes. The initial collision angle between the molecular axis and the CNT(15,0) surface was set as 45 degree. See Fig.3 (C,D,I,J).

File name: Supplementary Movie 5

Description: Real-time collision dynamics of ortho-2 showing the species-dependent delocalized nuclei and nodes. The initial collision angle between the molecular axis and the CNT(15,0) surface was set as 45 degree. See Fig.3 (C,D,I,J).
